# microRNA-146a inhibits G protein-coupled receptor-mediated activation of NF-κB by targeting CARD10 and COPS8 in gastric cancer

**DOI:** 10.1186/1476-4598-11-71

**Published:** 2012-09-20

**Authors:** Stephanie Geisler Crone, Anders Jacobsen, Birgitte Federspiel, Linda Bardram, Anders Krogh, Anders H Lund, Lennart Friis-Hansen

**Affiliations:** 1 Genomic Medicine, Rigshospitalet, University of Copenhagen, Rigshospitalet, Blegdamsvej 9, Copenhagen DK2100, Denmark; 2 Bioinformatics Centre, Institute of Molecular Biology, University of Copenhagen, Copenhagen, Denmark; 3 Computational Biology Center, Memorial Sloan-Kettering Cancer Center, New York, NY, USA; 4 Dept. of Pathology, Rigshospitalet, University of Copenhagen, Copenhagen, Denmark; 5 Dept. of Surgical Gastroenterology, Rigshospitalet, University of Copenhagen, Copenhagen, Denmark; 6 BRIC - Biotech Research & Innovation Centre and Centre for Epigenetics, University of Copenhagen, Copenhagen, Denmark

**Keywords:** Stomach cancer, Non-coding RNA, Cytokines

## Abstract

**Background:**

Gastric cancer is the second most common cause of cancer-related death in the world. Inflammatory signals originating from gastric cancer cells are important for recruiting inflammatory cells and regulation of metastasis of gastric cancer. Several microRNAs (miRNA) have been shown to be involved in development and progression of gastric cancer. miRNA-146a (miR-146a) is a modulator of inflammatory signals, but little is known about its importance in gastric cancer. We therefore wanted to identify targets of miR-146a in gastric cancer and examine its biological roles.

**Results:**

The expression of miR-146a was evaluated by quantitative PCR (qPCR) and found up-regulated in the gastrin knockout mice, a mouse model of gastric cancer, and in 73% of investigated human gastric adenocarcinomas. Expression of miR-146a by gastric cancer cells was confirmed by *in situ* hybridization. Global analysis of changes in mRNA levels after miR-146a transfection identified two transcripts, caspase recruitment domain-containing protein 10 (CARD10) and COP9 signalosome complex subunit 8 (COPS8), as new miR-146a targets. qPCR, Western blotting and luciferase assays confirmed these transcripts as direct miR-146a targets. CARD10 and COPS8 were shown to be part of the G protein-coupled receptor (GPCR) pathway of nuclear factor-kappaB (NF-kappaB) activation. Lysophosphatidic acid (LPA) induces NF-kappaB activation *via* this pathway and over-expression of miR-146a inhibited LPA-induced NF-kappaB activation, reduced LPA-induced expression of tumor-promoting cytokines and growth factors and inhibited monocyte attraction.

**Conclusions:**

miR-146a expression is up-regulated in a majority of gastric cancers where it targets CARD10 and COPS8, inhibiting GPCR-mediated activation of NF-kappaB, thus reducing expression of NF-kappaB-regulated tumor-promoting cytokines and growth factors. By targeting components of several NF-kappaB-activating pathways, miR-146a is a key component in the regulation of NF-kappaB activity.

## Background

Globally gastric cancer is the second most common cause of cancer-related death [1]. Development of gastric cancer is influenced by interactions between host, environmental and bacterial factors. Examples of synergistic risk factors for gastric cancer are polymorphisms in genes involved in the host inflammatory response [2], *Helicobacter pylori* (*H. pylori*) virulence factors [3] and diets rich in salt and nitrate [4]. Despite recent progress in detection and treatment of early gastric cancer, the long-term survival rate for advanced gastric cancer is low [5]. The main challenges in treatment of advanced gastric cancer are lymphatic, peritoneal or distant organ metastases, which simultaneously predict poor outcome for these patients [6].

Although many oncogenes and tumor suppressors have been reported to be involved in development of gastric carcinomas, the molecular mechanisms underlying metastasis of advanced gastric carcinomas are still poorly understood [7]. One of the key events in gastric carcinogenesis is inflammation. Inflammation leads to activation of the transcription factor nuclear factor-kappaB (NF-κB), which is associated with gastric carcinogenesis [8,9].

microRNAs (miRNA) are involved in the development and progression of gastric cancer [10-13]. miRNA is a class of endogenous, non-coding, single-stranded RNA molecules of app. 22 nucleotides that mediate post-transcriptional regulation of gene expression through base pairing with the 3’ untranslated region (UTR) of target messenger RNA (mRNA) [14]. miRNAs are involved in regulation of most cellular processes including cell proliferation, migration, differentiation and apoptosis [10,14,15]. miRNAs are aberrantly expressed in most human cancers and, like protein-coding genes, miRNAs can function as either tumor suppressors or oncogenes, thereby regulating carcinogenesis [10,12]. miRNA-146a (miR-146a) is regulated by NF-κB and inhibits interleukin-1 receptor (IL-1R) and toll-like receptor (TLR)- induced activation of NF-κB by targeting interleukin-1 receptor-associated kinase 1 (IRAK1) and TNF receptor associated factor 6 (TRAF6) [16-18]. miR-146a has been reported aberrantly expressed in several inflammatory diseases and cancers, but the role of miR-146a in gastric cancer is still controversial, as expression of miR-146a has been found both up- and down-regulated here [19-24]. Therefore, we investigated the expression of miR-146a in gastric cancer and characterized its targets and molecular functions to clarify the contradictory findings.

We found that miR-146a is up-regulated in a mouse model of gastric cancer as well as in human gastric adenocarcinomas and identified CARD10 and COPS8 as new direct targets of miR-146a. Both are part of the G protein-coupled receptor (GPCR)-mediated signal transduction that mediates activation of NF-κB. This suggests that miR-146a acts tumor suppressing by inhibiting GPCR-mediated activation of NF-κB and the resulting expression of tumor-promoting cytokines and growth factors.

## Results

### miR-146a expression is up-regulated in a mouse model of gastric cancer and in human gastric adenocarcinomas

**Figure 1:**
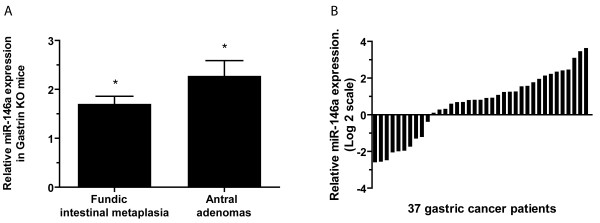
**Increased expression of miR-146a in the gastrin KO mouse gastric cancer model and human gastric cancer.** (***A***) The relative expression of miR-146a in metaplastic fundic tissue (n = 8) and hyperplastic antral mucosa (n = 4)/antral adenomas (n = 4) from gastrin knockout (KO) mice compared to the average expression in matching wild type (WT) mouse tissue. * = P < 0.05. (***B***) Waterfall plot of the expression of miR-146a in 37 human gastric adenocarcinomas relative to the expression in adjacent normal tissue. miR-146a was up-regulated in 27 out of 37 tumors (73%). The expression levels of miR-146a were determined by qPCR and normalized to those of the endogenous control RNU44.

**Figure 2:**
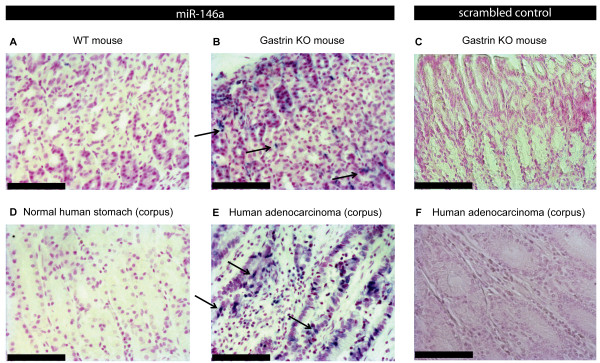
***In situ *hybridization detection of miR-146a expression in the gastrin KO mouse gastric cancer model and human gastric cancer.** (***A***) Expression of miR-146a was not detected in WT fundic mouse tissue, (***B***) while miR-146a-positive cells (blue nuclei) were detected in the metaplastic fundic tissue from gastrin KO mice. (***C***) No signal was detected with the scrambled LNA probe. (***D***) miR-146a expression was absent in normal human gastric tissue, (***E***) but present in human gastric adenocarcinoma cells (blue nuclei). (***F***) Negative control using a scrambled LNA probe. Representative miR-146a-positive cells are indicated by arrows. Original magnification x20, Scale bar = 100 μm.

Gastrin knockout (KO) mice are achlorhydric and develop intestinal metaplasia and gastric adenomas over time [25]. Using quantitative PCR (qPCR) we found the expression of miR-146a app. 2-fold up-regulated in old gastrin KO mice with either fundic intestinal metaplasia or antral adenoma compared to the expression in wild type (WT) mice (P < 0.05) (Figure 1A). Using *in situ* hybridization miR-146a was detected in metaplastic gastric tissue from the gastrin KO mice, but not in normal gastric tissue from the WT mice (Figure 2AB) (Additional file 1: Figure S1). Having established that miR-146a is increased in our mouse model of gastric cancer we examined expression of miR-146a in paired human gastric adenocarcinomas and adjacent control biopsies and found that it was up-regulated in 27 out of 37 cases (73%) (Figure 1B). *In situ* hybridization showed that miR-146a was expressed by the human gastric adenocarcinoma cells, while miR-146a-positive cells were not detected in the normal gastric mucosa (Figure 2CD) (Additional file 1: Figure S1).

There was no correlation between miR-146a expression in gastric adenocarcinomas and patients’ age, sex and localization or classification of tumors (data not shown). Although patients with high miR-146a expression seemed to have a better overall survival this was not significant ( Additional file 1: Figure S2).

### miR-146a targets members of the GPCR-mediated NF-κB activation pathway

Having demonstrated increased expression of miR-146a in the majority of gastric cancers, we wanted to establish the biological actions of miR-146a by characterizing its direct molecular targets in human gastric cancer. We wanted to do this by over-expressing miR-146a in gastric cancer cells and then identifying mRNAs with reduced expression [26]. Therefore, we examined miR-146a expression in a panel of cell lines and found varied, but surprisingly low expression of miR-146a in the available gastric cell lines (Additional file 1: Figure S3), considering the detected over-expression in tumors.

The human gastric cancer cell line SNU638, which has neglectable levels of endogenous miR-146a was found suited for miR-146a over-expression studies. Since miR-146a expression was very low in the tested gastric cell lines miR-146a inhibition studies were not conducted.

**Figure 3:**
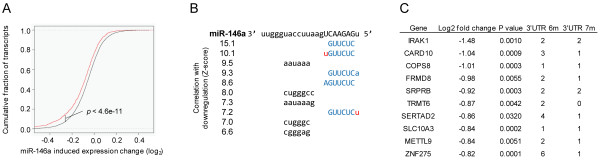
**Identification of miR-146a targets.** (***A***) Predicted miR-146a target transcripts are significantly down-regulated after miR-146a transfection compared to expressed genes that are not predicted targets of miR-146a (P < 4.6e-11, two-tailed Wilcoxon rank-sum test). (***B***) Top 10 words most correlated with down-regulation (correlation Z-scores are indicated to the left of each word, all 10 words have an estimated FDR < 1e-6). Capital letters highlight the miRNA seed region, Watson-Crick base-pairs (blue), mismatches (red). The word strongest correlated with down-regulation was the seed site complementary to mature miRNA bases 2-7/8. (***C***) Transcripts, having 6mer or 7mer miR-146a seed sites in the 3’UTR, that are most down-regulated upon miR-146a transfection are shown. These are potential direct miR-146a targets. Expression is given in log2 fold change. The 3’UTR 6 m/7 m columns indicate numbers of 6- or 7mer miR-146a seed sites in the 3’UTR.

We first tested if over-expression of miR-146a affected the growth of the SNU638 cells and found cell growth unaffected (Additional file 1: Figure S4). Subsequently, global changes in gene expression in SNU638 cells following over-expression of miR-146a were examined. After miR-146a transfection mRNAs with predicted 3’UTR miR-146a target sites were significantly down-regulated compared to mRNAs without predicted targets sites (P < 4.6e-11, two-tailed Wilcoxon rank-sum test) (Figure 3A). We analyzed all words of length 5–7 for over-representation in down-regulated mRNAs after miR-146a transfection and found the word strongest correlated with down-regulation was the seed site complementary to mature miR-146a bases 2-7/8 (Figure 3B). Transcripts with predicted 3’UTR miR-146a target sites that were significantly down-regulated upon miR-146a transfection were regarded as potential direct miR-146a targets. 847 matched these criteria (Additional file 1: Table S5). The top 10 most down-regulated potential miR-146a targets are shown in Figure 3C. As a negative validation control we repeated the procedure treating SNU638 cells with a miR-146a LNA inhibitor. There was no significant up-regulation of genes with the seed site complementary to mature miR-146a bases (data not shown).

The three most down-regulated genes upon miR-146a over-expression, IRAK1, caspase recruitment domain-containing protein 10 (CARD10) and COP9 constitutive photomorphogenic homolog subunit 8 (COPS8) (Figure 3C) all belong to signaling pathways leading to NF-κB activation [27-29]. IRAK1 is a known miR-146a target involved in TLR- and IL-1R-mediated activation of NF-κB [16-18]. CARD10 is involved in GPCR-mediated activation of NF-κB [27], while COPS8 is thought to be involved in this pathway based on its involvement in T cell receptor (TCR)-mediated NF-κB activation [28]. Surprisingly, but similar to the findings of Boldin *et al.*[30], expression of TRAF6, which has previously been described as a miR-146a target [16,18,31], was not reduced at the transcriptional level after miR-146a over-expression in our model system.

**Figure 4:**
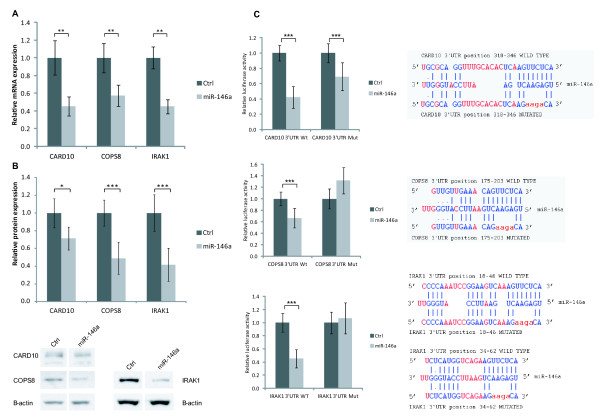
**CARD10 and COPS8 are direct miR-146a targets.** (***A***) mRNA expression of the two new miR-146a targets, CARD10 and COPS8, and IRAK1, a known miR_146a target, was determined by qPCR in control- and miR-146a-transfected SNU638 cells. Expression of each transcript is shown relative to the expression in control-transfected cells. CARD10, COPS8 and IRAK1 expression was down-regulated following miR-146a over-expression. Data are shown as mean ± S.D. of four biological replicates. * = P < 0.05, ** = P < 0.01, *** = P < 0.001. (***B***) miR-146a transfection also reduced the protein levels of CARD10, COPS8 and IRAK1 in miR-146a-transfected SNU638 cells. The expression of each protein is shown relative to the expression in control-transfected cells. Bands were quantified relative to β-actin. Data are shown as mean ± S.D. of four biological replicates. * = P < 0.05, ** = P < 0.01, *** = P < 0.001. (***C***) Verification of direct and functional target binding using luciferase constructs holding WT 3’UTRs and mutated (Mut) 3’UTRs (the central 4 nucleotide were replaced in miR-146a binding site) for CARD10, COPS8 and IRAK1. Constructs containing either WT or mutated (Mut) 3’UTRs downstream to the firefly luciferase gene were co-transfected into HEK293 cells together with Renilla luciferase control plasmid and either miR-146a or control (siGlo). Left, luciferase activity is given relative to activity in control-transfected cells. miR-146a reduced luciferase activity of constructs with WT CARD10, COPS8 and IRAK1 3’UTRs. Right, sequence alignments of miR-146a and potential WT and Mut 3’UTR target sites are shown, mutated bases are shown in red. Data are shown as mean ± S.D. often biological replicates. *** = P < 0.001. Sequence alignments are adapted from DIANALab (2009).

In gastric cancer, NF-κB modulates cell survival, immunity and inflammation, and NF-κB activation is associated with poor outcome in gastric cancer [32,33]. We therefore focused on characterizing CARD10 and COPS8 as direct miR-146a targets and their roles in NF-κB activation in gastric cancer. We confirmed miR-146a-mediated down-regulation of CARD10, COPS8 and IRAK1 at the transcript level (Figure 4A) and also found that miR-146a reduced levels of CARD10, COPS8 and IRAK1 protein (Figure 4B). Finally, direct targeting of miR-146a to 3’UTRs of the target genes was demonstrated using luciferase assays (Figure 4C). In summary, we confirmed earlier observations showing that miR-146a directly targets IRAK1 and we furthermore identified two new targets, CARD10 and COPS8, which codes for proteins suggested to be involved in NF-κB activation. COPS8 is a component of the COP9 signalosome which consists of eight subunits (GPS1 and COPS2-8) [34]. COPS8 is the only subunit targeted directly by miR-146, but since alteration in the amount of the individual subunits has been shown to affect the amount of other subunits [35,36], we examined how transfection with miR-146a affected expression of all COP9 signalosome components. In unstimulated cells the expression of COPS2 was reduced (20%) (Additional file 1: Figure S5). We therefore assume that the effects of miR-146a on the COP9 complex mainly result from a reduction in COPS8 expression, although indirect destabilization of the complex cannot be ruled out.

### miR-146a inhibits GPCR-mediated NF-κB activity by targeting CARD10 and COPS8

**Figure 5:**
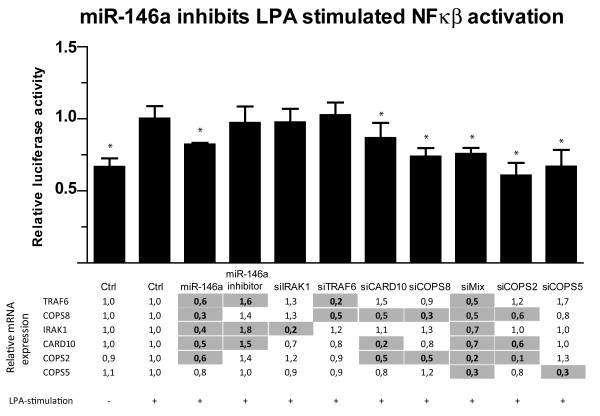
**miR-146a targets COPS8 and CARD10 GPCR-mediated NF-κB activation.** SNU638 cells were co-transfected with NF-κB reporter plasmid and Renilla luciferase control plasmid along with control (siGlo), siRNA or miR-146a. Cells were stimulated with 25 μM LPA and luciferase activity was measured. Luciferase activity is given relative to activity in control transfected LPA-stimulated cells. siRNA against CARD10 and COPS8, miR-146a, miR-146a inhibitor and a siRNA mix reduced luciferase activity. siRNA against IRAK1 and TRAF6 did not reduce luciferase activity. LPA-stimulated NF-κB activation was also inhibited by siRNA against COPS2 and COPS5, demonstrating that other perturbation of the COP9 signalosome complex reduces GPCR-mediated NF-κB activation. siMix, mix of siRNAs against CARD10, COPS8, IRAK1 and TRAF6. Data are shown as the mean ± S.D. of seven biological replicates.* = P < 0.05. The relative expression of TRAF6, COPS8, IRAK1, CARD10, COPS2 and COPS5 was measured using qPCR. Gray-shading indicates a significant change in expression, p < 0.05, n = 4.

CARD10 and COPS8 are involved in GPCR-mediated activation of NF-κB [28,29]. We therefore wanted to establish their roles in signal transduction in gastric cancer and subsequently investigate the importance of miR-146a for inhibiting this signaling. For this purpose we used lysophosphatiditc acid (LPA) which is a known activator of the GPCR-mediated NF-κB-activation pathway [29], and promotes gastric cancer cell migration and invasion [37]. LPA-stiumlation significantly increased NF-κB activity in our luciferase reporter system (Figure 5). siRNA knockdown of CARD10 and COPS8 expression significantly inhibited LPA-stimulated GPCR-mediated activation of NF-κB in SNU638 cells (Figure 5). This inhibition was comparable to the miR-146a-induced inhibition. In contrast, inhibiting endogenous miR-146a was without effect on NF-κB activation. As predicted, siRNA-mediated repression of IRAK1 expression did not affect LPA-stimulated activation of NF-κB (Figure 5) as IRAK1 is not involved the GPCR-mediated pathway. As TRAF6 is a miR-146a target with a role in NF-κB activation, the effect of siRNA-mediated repression of TRAF6 expression on LPA-stimulated NF-κB activity was investigated. However, knockdown of TRAF6 did not inhibit LPA-stimulated NF-κB activity (Figure 5). These results indicate that the two newly identified miR-146a targets, CARD10 and COPS8, are involved in GPCR-mediated activation of NF-κB. Therefore, the effect of miR-146a over-expression on LPA-stimulated NF-κB activity was studied. miR-146a significantly inhibited LPA-stimulated NF-κB activity in SNU638 cells (Figure 5). A mix of siRNAs against CARD10, COPS8, IRAK1 and TRAF6 mimicked the miR-146a-mediated inhibition of NF-κB activity (Figure 5). Knockdown of two other COP9 components (COPS2 and COPS5) also inhibited the LPA-stimulated NF-κB activity, demonstrating the importance of presence of all COP9 signalosome subunits for LPA-stimulated activation of NF-κB. qPCR of the expression of the different components examined confirmed the knockdown (Figure 5).

The expression of miR-146a is in part controlled by NF-κB. We therefore also tested how the expression of miR-146a was affected by LPA and IL-1β stimulation. We found that LPA treatment doubled the expression of miR-146a and IL-1β stimulation gave a 4-fold increase in miR-146a expression, which is comparable to earlier observations [16,38] (Additional file 1: Figure S6). Although miR-146a LNA increased the expression of three of the miR-146a targets (TRAF6, IRAK1 and CARD10) (Figure 5) the overall activation of NF-κB was not altered in LPA-stimulated miR-146a LNA treated SNU638 cells.

### miR-146a reduces LPA-induced expression of cytokines and growth factors

**Figure 6:**
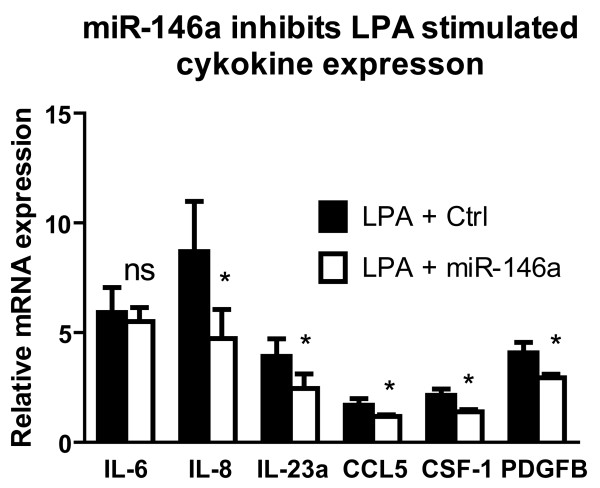
**miR-146a inhibits LPA-induced cytokine expression.** 25 μM LPA was used to induce cytokine expression from human gastric carcinoma SNU638 cells. LPA significantly enhanced expression of IL-6, IL-8, IL-23A, CCL5, CSF-1 and PDGFB, which was measured using qPCR and shown relative to the expression level in untreated control cells. This response is reduced in miR-146a-transfected cells. Data are shown as mean ± S.D., n = 4, * = P < 0.05, ns = non significant.

In most carcinomas microenvironmental factors rather than genetic alterations are probably responsible for activating NF-κB signaling that regulates several processes including secretion of growth factors and cytokines [39,40]. Therefore, we investigated how miR-146a modulates expression of selected NF-κB-regulated growth factors and cytokines induced by extracelluar signals such as LPA. LPA-stimulation significantly increased expression of interleukin-6, -8, 23A (IL-6, IL-8, IL-23A) and chemokine (C-C motif) ligand 5 (CCL5), colony stimulating factor 1 (macrophage) (CSF-1) and platelet-derived growth factor beta polypeptide (PDGFB) in SNU638 cells (Figure 6). This confirmed that expression of these genes is regulated by LPA-induced NF-κB activity. Over-expression of miR-146a significantly decreased expression of IL-8, IL-23A, CCL5, CSF-1 and PDGFB in SNU638 cells (Figure 6). Even though IL-6 is a target gene of NF-κB, and also upregulated by LPA miR-146a over-expression did not reduce IL-6 expression under our conditions (Figure 6). However, LPA induction of IL-6 expression is not only mediated by NF-κB but also via other pathways (MAPK/ERK, PI3K/Akt, and p38) [41], which may contribute to the differences. In SNU638 cells the basal level of IL-6 is higher than IL-8, which may affect the mRNA turnover. This could be part of the reason that miR-146a has less effect on LPA-stimulated IL-6 expression than on IL-8 expression, which has also been seen in other cellular systems [42].

### miR-146a over-expression in SNU638 cells reduces recruitment of monocytes

**Figure 7:**
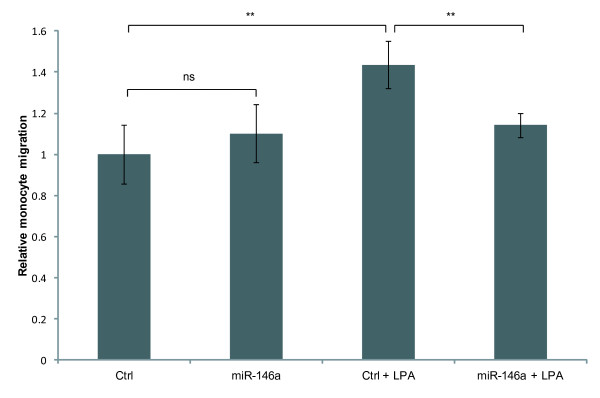
**miR-146a reduces monocyte migration.** Monocyte migration against conditioned medium from control- or miR-146a-transfected SNU638 cells that were left untreated or stimulated with 25 μM LPA was followed over 8 hours. Migration of cells is shown relative to migration of untreated control cells. Monocyte migration against medium from LPA-stimulated SNU638 cells was increased compared to migration against medium from untreated cells, while monocyte migration against medium from miR-146a-transfected, LPA-stimulated cells was decreased compared to medium from control-transfected, LPA-stimulated cells. Data are shown as mean ± S.D. of four biological replicates. ** = P < 0.01, ns = non significant.

In carcinomas monocytes can be recruited by e.g. CCL5 and CSF-1 expressed by tumor cells and this tumor infiltration of monocytes contributes to the tumor-promoting inflammatory response in the cancer [39,40]. Having demonstrated that miR-146a reduced LPA-induced expression of cytokines, we examined how miR-146a over-expression affected recruitment of monocytes. We found that LPA-treatment of SNU638 cells increased monocyte migration towards conditioned medium from the SNU638 cells (Figure 7 and Additional file 1: Figure S7), whereas transfection with miR-146a in part abrogated this response (Figure 7), again demonstrating that miR-146a inhibits the biological responses of GPCR-mediated activation of NF-κB such as, recruitment of monocytes.

## Discussion

Microenvironmental factors are important for NF-κB activation in inflammation-driven cancers such as gastric cancer and this NF-κB activation may provide cancer cells with advantages, which contribute to the tumorgenic processes [32,43]. Modulation of NF-κB-activating signaling is therefore important for controlling inflammation-mediated tumor development. Here, we show that miR-146a is a central negative regulator of NF-κB activation as it inhibits several NF-κB-activating pathways.

miR-146a expression was found up-regulated in app. 2/3 of the human gastric adenocarcinomas as well as in the gastrin KO mouse model of gastric cancer. Similarly, expression of miR-146a has been found increased in cervical, breast, pancreatic and thyroid cancer [11,44-46]. Previously, expression of miR-146a has been found both up- and down-regulated in gastric cancer [21-24]. These conflicting results may reflect differences in tumors and their microenvironment resulting in different degrees of NF-κB activities, which controls miR-146a expression [16]. Surprisingly, we found low levels of miR-146a in human gastric cancer cell lines. This could indicate that the increased miR-146a levels seen in tumors are coursed by microenvironmental factors e.g. cells surrounding the tumor cells.

To examine the effects of increased miR-146a levels in gastric cancer we identified two new miR-146a targets, CARD10 and COPS8, and investigated the roles of these targets. We found that miR-146a inhibited GPCR-mediated NF-κB activation by directly targeting and down-regulating expression of CARD10 and COPS8. CARD10 is known to be required for GPCR-induced activation of NF-κB [27,29], while COPS8 is a subunit of COP9 signalosome that controls NF-κB activation [28,47]. We showed that CARD10 is involved in GPCR-mediated activation of NF-κB, and provided new evidence that COPS8 is involved in this pathway as well. miR-146a over-expression also led to reduced expression of COPS2, another subunit of the COP9 signalosome. We assume that this is an indirect effect, since there is no miR-146a binding site in COPS2 3’UTR and changes in the expression of one subunit has been shown to affect that of the other [35,36]. It is therefore possible that miR-146a to some extent may act by not only targeting COPS8 but destabilizing the COP9 signalosome.

**Figure 8:**
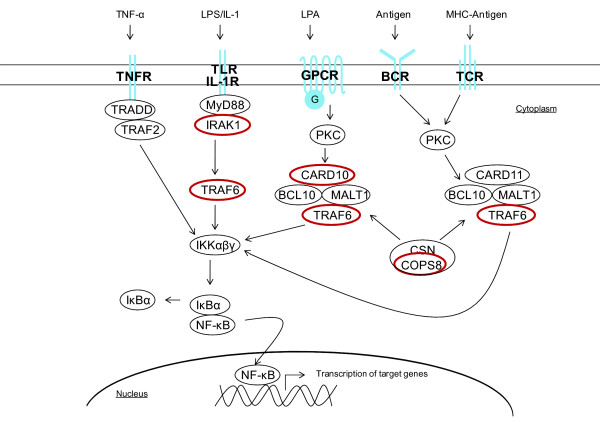
**miR-146a targets multiple NF-κB activation pathways.** Schematic illustration of NF-κB-activating pathways. miR-146a has previously been shown to target IRAK1 and TRAF6 and in this study we show that miR-146a targets CARD10 and COPS8. miR-146a targets are marked with red circles.

Aberrant signaling through GPCRs has been linked to tumor cell growth and survival, and NF-κB-activating GPCRs have been shown to contribute to a wide range of cancers (reviewed by Dorsam & Gutkind [48]). GPCRs can activate NF-κB *via* the CARD10/BCL10/MALT1 complex after binding ligands such as LPA, enothelin-1, angiotensin II (Ang II) and chemokine (C-X-C motif) ligand 12 (CXCL12) (Figure 8) [29,49]. In our study we used LPA to model GPCR-mediated activation of NF-κB. LPA-induced GPCR-signaling leads to tumor progression [49]. Thus, miR-146a-mediated inhibition of GPCR-signaling could have tumor suppressing effects.

In addition to GPCR-mediated NF-κB activation, NF-κB can be activated *via* tumor-necrosis-factor receptor (TNFR), IL-1R, TLR, TCR and B cell receptor (BCR) [50] (Figure 8). Previously, miR-146a has been shown to inhibit NF-κB activation *via* these receptors by down-regulating expression of TRAF6 and IRAK1 [16,18,30,31]. miR-146a targets IRAK1 in gastric cancer cells, but not TRAF6. Although, we here show that miR-146a targets GPCR-mediated activation of NF-κB, in addition to the activation *via* TNFR, IL-1R, TLR, TCR and BCR, by targeting CARD10 and COPS8 (Figure 8). Thus, miR-146a targets multiple components of NF-κB-activating pathways in gastric cancer cells. This has not been shown for the NF-κB pathway before, but has previously been seen in the transforming growth factor-β (TGF-β) pathway, where both miR-21 and the miR-17-92 cluster target several mRNAs coding for proteins in the TGF-β pathway [51,52]. By targeting multiple components of different NF-κB-activating pathways miR-146a mediates a robust and complex control of NF-κB activity. Several other miRNAs can also regulate NF-κB-signaling, but only miR-146a targets several genes in different NF-κB-activating pathways, suggesting miR-146a as a key modulator of NF-κB activation.

**Figure 9:**
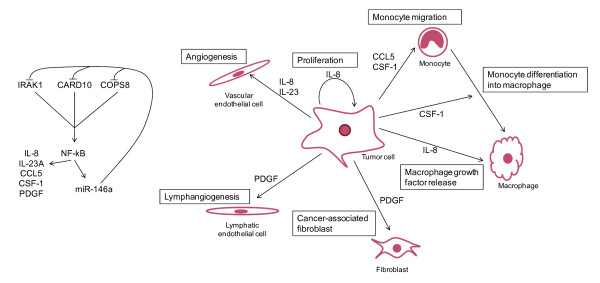
**Biological processes in gastric cancer cells inhibited by miR-146a.** Left panel, miR-146a directly targets IRAK1, CARD10 and COPS8 in SNU638 cells. This leads to inhibition of NF-κB activation and consequently to repressed expression of NF-κB-regulated cytokines and growth factors. Expression of miR-146a is also regulated by NF-κB [16]**.** Right panel, miR-146a decreases expression of CCL5, CSF-1, IL-8, PDGF and IL-23A, which are involved in monocyte migration and differentiation, proliferation, angiogenesis, lymphangiogenesis and fibroblast and macrophage growth factor release. Thus, miR-146a might act as a tumor suppressor by reducing cancer-promoting effects of NF-κB activity.

NF-κB regulates expression of cytokines and growth factors involved in several aspects of tumor progression [43]. Given that miR-146a decreases NF-κB activity, it is possible that miR-146a acts tumor suppressing by reducing expression of such cytokines and growth factors. Indeed, we found that miR-146a over-expression reduced expression of several cytokines and growth factors with a known role in cancer development; IL-8, IL-23A, CCL5, CSF-1 and PDGFB. These genes can increase cell proliferation, cell adhesion and angiogenesis [53-55], contribute to tumor lymphangiogenesis [56], activate fibroblasts [57], recruit monocytes to tumors [39,40] and induce tumor-associated macrophages (TAMs) to secrete tumor-promoting mediators [58] (Figure 9).

Chemotactic cytokines released by tumor cells can recruit monocytes to tumor sites [39,59]. These monocytes can promote tumor progression [60]. CCL5 and CSF-1 are examples of monocyte-recruiting cytokines released by tumor cells [39,40]. As expected, monocytes migrated less against conditioned medium from LPA-stimulated SNU638 cells over-expressing miR-146a compared to LPA-stimulated control cells. Thus, miR-146a may reduce tumor infiltration of monocytes by decreasing tumor cell expression of cytokines.

Up-regulated levels of miR-146a in gastric cancer seen in this study could be caused by increased NF-κB activity in tumor cells. miR-146a is part of a negative feedback loop that inhibits NF-κB activation in gastric cancer and the subsequent tumor-promoting processes. This is supported by a recent study that found low expression of miR-146a associated with poor survival of gastric cancer patients [22].

## Conclusions

In summary, we have identified two new targets of miR-146a, CARD10 and COPS8 that are both involved in GPCR-mediated activation of NF-κB, and we have found that miR-146a inhibits secretion of chemokines and growth factors controlled by NF-κB. With the addition of two new miR-146a targets we have shown that this miRNA targets several signal transduction pathways that activate NF-κB. Hence, we suggest that miR-146a act tumor-suppressing by inhibiting NF-κB activity and the consequently expression of tumor-promoting cytokines and growth factors.

## Methods

### Mice

Groups of WT and gastrin KO mice aged 1½ years were used. The mice were on a mixed 129/SvJ, C57BL/6J background backcrossed at least four times to C57BL/6J [25]. The mice were sacrificed by cervical dislocation. The stomachs were removed, washed gently in ice-cold PBS and the fundus was dissected from the stomach, frozen in liquid nitrogen and stored at −80°C until RNA extraction. The mice were kept under specific pathogen-free conditions and monitored according to the Federation of European Laboratory Animal Science Associations recommendation [61]. The studies were approved by the Danish Animal Welfare Committee (2005/562-40) and the Danish Forest and Nature Agency (20010077355/6).

### Human tissue samples

Biopsies from human gastric adenocarcinomas and adjacent normal tissue were obtained from patients who underwent surgical resection at the Department of Gastrointestinal Surgery, Rigshospitalet, Copenhagen, Denmark, between July and December 2008. Collection of gastric cancer biopsies was approved by the Danish Ethical Committee (journal H-B-2008-049) and the establishment of a biobank was approved by Danish Data Protection Agency (journal 2008-41-2138). All procedures were in accordance with institutional ethical standards. All individuals provided written informed consent, and all samples were delinked and unidentified from their donors

### Cell culture and transfections

SNU638 cells were grown in RPMI1640 medium (Invitrogen, Carlsbad, CA) and HEK293 cells in DMEM + GlutaMAX™-I (Invitrogen), both supplemented with 10% Fetal Bovine Serum (Biowest, Nuaillé, France), penicillin (100 U/ml), streptomycin (100 μg/ml) (Sigma-Aldrich, Saint Louis, MI) and cefotaxim (100 μg/ml) (ACS Dobfar Generics S.A., Luxembourg, Belgium). Cells were cultured at 37°C in 5% CO_2_. Where nothing else is stated cells were transfected using Turbofect™ *in vitro* Transfection Reagent (Fermentas GmbH, St. Leon-Rot, Germany). To over-express miR-146a in cultured cells 50 nM Pre-miR™ miRNA Precursors for human miR-146a (hsa-miR-146a, Ambion, Invitrogen) were used and 50 nM siGlo (Dharmacon, Lafayette, CO) were used as negative control.

### RNA isolation

Where nothing else is stated total RNA from tissue and cells was isolated using TRIzol® Reagent (Invitrogen).

### miR-146a over-expression analysis

For mRNA microarrays SNU638 cells were transfected with miR-146a or siGlo using Lipofectamine2000 (Invitrogen). 24 h post-transfection total RNA was isolated. Affymetrix microarray analysis was performed at the Microarray Center, Rigshospitalet, Copenhagen, Denmark, using HG-U133 Plus 2.0 human arrays (Affymetrix, Santa Clara, CA) as previously described [62] and predicted miR-146a target genes were identified [63]. Briefly, 2 μg of total RNA was used to synthesize double-stranded cDNA using Superscript Choice System (Invitrogen) with an oligo(dT) primer containing a T7 RNA polymerase promoter. Subsequently, cDNA was used as template for an *in vitro* transcription reaction generating biotin-labeled antisense cRNA (BioArrayTM High Yield RNA Transcript Labeling kit; Enzo Diagnostics, Farmingdale, NY). Arrays were scanned in an Affymetrix GeneArray® 2500 scanner and data were analyzed using D-chip MFC Application (software version 1.0.0.1). Three biological replicates of each transfection were analyzed. The microarray expression data were processed using the 'affy' package in BioConductor. Probeset intensities were summarized and quantile normalized using the RMA and VSN packages. Differential expression was determined per probe set using a t-test. The probe sets were mapped to Ensembl transcripts (version 49) using mappings obtained from BioMart. Probe sets that mapped ambiguously to different Ensembl genes were discarded. 3’UTRs were scanned for matching 6mer, 7mer and 8mer miR-146a target sites (complementary to position 2–7, 2–8, and 2–9 of the miRNA sequence). To evaluate if predicted miR-146a targets were down-regulated after miR-146a transfection, we tested the null hypothesis that the expression change distribution of predicted miR-146a target genes (having a 7mer target site in the 3’UTR) was identical to the distribution of all expressed genes without predicted target sites using the non-parametric Wilcoxon rank-sum test. We used a previously published non-parametric rank-based statistic to perform an exhaustive and unbiased assessment of the correlation of 3’UTR word occurrences and the change in gene expression after miR-146a transfection [63]. Genes were sorted by their expression change after miR-146a transfection, and the correlation with down-regulation was tested for all words of length 5–7 (N = 21504).

### Quantitative PCR

Mature miR-146a expression was measured and quantified using TaqMan® MicroRNA Assay hsa-miR-146a (Applied Biosystems, Foster City, CA). For each biological sample technical triplicates were made. Analyses were carried out on the ABI Prism® 7900HT Sequence Detection System (SDS) (Applied Biosystems). miR-146a levels were normalized to the endogenous control RNU44 (Applied Biosystems).

For mRNA qPCR, total RNA from transfected cells was isolated and qPCR was performed using SuperScript™ III First-Strand Synthesis SuperMix (Invitrogen) and SYBR GreenER™ qPCR SuperMix for ABI PRISM® (Invitrogen). The analyses were carried out on ABI Prism® 7900HT SDS (Applied Biosystems). Primers were designed using primer3 (Additional file 1: Table S1) or predesigned primers (Quantitect, Qiagen, Hilden, Germany) were used. For each biological sample technical triplicates were made. Expression of target mRNA was normalized to GAPDH expression and quantified using standard curves.

### *In situ* hybridization

Gastric tissues from WT and gastrin KO mice were formalin-fixed and paraffin-embedded (FFPE). FFPE tissue samples of human gastric adenocarcinomas and normal gastric tissues were obtained from the Department of Pathology, Rigshospitalet, Copenhagen, Denmark. A DIG-labeled mercury locked nucleic acid miR-146a detection probe (Exiqon, Vedbæk, Denmark) was used for detection as described by Jørgensen *et al.*[64]. Probe concentration was 100 nM and slides were hybridized at 50°C. Pictures of representative areas of the slides were taken with a Zeiss Axio Imager (Zeiss, Jena, Germany), original magnification x20/10. Cells with intense blue nuclear stain were scored as positive. The level of expression within a positive cell was not scored. A LNA probe against snRNA U6 (Exiqon) was used as positive control and a scramble probe (Exiqon) as negative control.

### Western blotting

For Western blotting SNU638 cells were transfected with miR-146a or siGlo and cells were harvested 6 and 72 h post-transfection. Proteins were separated on polyacrylamide gels, transferred to nitrocellulose membranes, incubated with antibodies against IRAK1 (4359, Cell Signaling Technology, Danvers, MA), CARD10 (ab64171, abcam, Cambridge, UK), COPS8 (ab77300, abcam) or β-actin (A2228, Sigma-Aldrich) and visualized by chemiluminescence (SuperSignal® West Pico, Pierce, Rockford, IL) using LAS-1000 Pro v. 2.6 (FujiFilm, Tokyo, Japan). Protein band intensities were quantified using Multi Gauge Software v. 3.1 (FujiFilm). β-actin was used as loading control.

### 3’UTR luciferase assay

3’UTR luciferase reporter plasmids were constructed by amplifying CARD10 and COPS8 3’UTR fragments containing potential miR-146a binding sites from human genomic DNA (primer sequences in Additional file 1: Table S2). Fragments were cloned into pMIR-REPORT Luciferase miR Expression Reporter Vector (Applied Biosystems) downstream of the Firefly luciferase gene. miR-146a seed sites were mutated by substitution of four nucleotides using QuickChange Site-Directed Mutagenesis Kit (Agilent Technologies, Palo Alto, CA), thereby changing the sequence from AGTTCTCA to AGAAGACA (mutagenesis primers in Additional file 1: Table S3). pMIR-REPORT plasmids containing WT and mutated IRAK1 3’UTR site were made by Taganov *et al.*[16] (Addgene plasmids 15095/15096). HEK293 cells were plated at 1x10^5^ cells/well in 24-well plates and transfected 24 h later. HEK293 cells were used as they generally have low endogenous miRNA levels [65]. Each transfection reaction contained 10 ng luciferase pMIR-REPORT and 20 ng Renilla vector (pRL-TK, Promega, Madison, WI) together with 50 nM miR-146a (Ambion) or siGlo (Dharmacon). 24 h post-transfection Firefly luciferase and Renilla luciferase luminescence was measured using Dual-Glo luciferase kit (Promega) and a GloMax®-96 luminometer (Promega). Background measurements were subtracted and ratios of Firefly luciferase luminescence from pMIR-REPORT relative to Renilla luciferase luminescence from pRL-TK were calculated.

### NF-κB activity assay

SNU638 cells were plated at 1x10^5^cells/well in 24-well plates and transfected after 24 h. Each transfection reaction contained 500 ng NF-κB luciferase reporter plasmid [66], 50 ng pRL-TK (Promega) and 50 nM siGlo (Dharmacon), 50 nM miR-146a (Ambion), 50 nM miCURY miR-146a inhibitor (Exiqon) or 50 nM siRNAs against CARD10, COPS8, IRAK1 or TRAF6 (Flexitube, Qiagen, Hilden, Germany). 24 h post-transfection cells were stimulated with 25 μM LPA (Avanti Polar Lipids, Alabaster, AL). 24 h after stimulation Firefly luciferase and Renilla luciferase luminescence was measured as described above. Background measurements were subtracted and ratios of luminescence from NF-κB reporter plasmid relative to luminescence from pRL-TK were calculated.

### Monocyte migration

Monocytes were isolated by density gradient centrifugation followed by plastic adherence. Peripheral blood mononuclear cells were isolated from blood from healthy donors by density centrifugation with Lymphoprep using a standard protocol (Axis-Shield Poc AS, Oslo, Norway) [67]. Cells were plated in plastic dishes and allowed to adhere for 1 h. Non-adherent cells were washed away and adherent monocytes were used for migration studies. Monocytes were seeded in the upper chambers of CIM-plate 16 (Roche Diagnostics GmbH, Manheim, Germany). 8x10^5^ cells/well were seeded in RPMI1640 medium containing 1%. Lower chambers contained conditioned medium from siGlo- or miR-146a-transfected SNU638 cells that were left untreated or treated with 25 μM LPA (Avanti Polar Lipids) for 6 hours. Migration was followed real-time over 8 hours with xCELLigence impedance analysis using the RTCA DP instrument (Roche Diagnostics GmbH). This method allows continuous measurement of cell migration by measuring the electrical impedance over gold electrodes incorporated on the underside of a microporous polyethylene terephthalate dividing an upper and lower. Migration rates were calculated using the RTCA software (Roche Diagnostics GmbH).

### Statistical analysis

Where nothing else is stated statistical analyses were performed using Student’s unpaired two-tailed t-test calculated by Excel’s ToolPak or GraphPad Prism Software. P-values less than 0.05 were considered significant. The patient overall survival from the day of surgery was examined using the Kaplan-Meier method, with log-rank test (Mantel-Cox test) and the Gehan-Breslow-Wilcoxon test for statistical significance.

## Abbreviations

BCR: B cell receptor; CARD10: Caspase recruitment domain-containing protein 10; CCL: Chemokine (C-C motif) ligand; COPS8: COP9 signalosome complex subunit 8; CSF-1: Colony stimulating factor 1 (macrophage); FFPE: Formalin-fixed and paraffin-embedded; GPCR: G Protein-coupled receptors; IL: interleukin; IL-1R: Interleukin-1 receptor; IRAK1: interleukin-1 receptor-associated kinase 1; KO: knockout; LPA: Lysophosphatidic acid; miR-146a: microRNA-146a; miRNA: microRNA; mRNA: Messenger RNA; NF-κB: nuclear factor-kappaB; PDGFB: Platelet-derived growth factor beta polypeptide; TAM: Tumor-associated macrophages; TCR: T cell receptor; TGF: Transforming growth factor; TLR: Toll-like receptor; TNFR: Tumor-necrosis-factor receptor; TRAF6: TNF receptor associated factor 6; UTR: Untranslated region; WT: Wild type.

## Competing interests

The authors declare that they have no competing interests.

## Authors’ contributions

Study concept and design: SGC, AHL, AK, LFH. Acquisition of data: SGC, AJ, BF, LB, LFH. Analysis and interpretation of data: SGC, AJ, AK, LFH. Drafting of the manuscript: SGC, LFH. Critical revision of the manuscript for important intellectual content: SGC, AK, AHL, LB, AJ, LFH. Statistical analysis: SGC, AJ. Obtained funding: SGC, LFH. Administrative, technical, or material support: SGC, BF, LB, LFH. Study supervision: LFH, AHL. All authors read and approved the final manuscript.

## Supplementary Material

Additional file 1 **Figure S1.** Expression of miR-146a in the gastrin KO mice and in human gastric cancer. *In situ* hybridization detection of miR-146a expression. (*A* and *B*) miR-146a expression was absent in WT fundic mouse tissue, while miR-146a-positive cells (blue nuclei) were detected in the metaplastic fundic tissue from gastrin KO mice. (*C* and *D*) miR-146a expression was absent in normal human gastric tissue, while human gastric adenocarcinoma cells stained for miR-146a (blue nuclei). Original magnification x10, Scale bar = 100 μm. (PDF 1343 kb). **Figure S2.** Survival of gastric cancer patients with low or high tumor miR-146a expression. Kaplan-Meier overall survival curves according to miR-146a level. Although patients whose tumors have high expression of miR-146a seemed to have better survival than those with low expression, it was not significant, p = 0,31 (Mantel-Cox Test). Low expression was defined as those with a relative miR-146a expression below the median expression and high expression had relative expression above median. **Figure S3.** Relative expression of miR-146a in cell lines. The expression of miR-146a in cells lines determined by qPCR. Gastric cancer cell lines are indicated by red bars. Relative miR-146a expression was determined using TaqMan® MicroRNA Assay hsa-miR-146a according to the manufacturer’s protocol (Applied Biosystems). miR-146a levels were normalized to hsa-miR-191 (Applied Biosystems), which served as an endogenous control. **Figure S4.** Normal growth of SNU638 cells transfected with miR-146a in cell lines. 2,5 106 SNU638 cells were seeded in 10 cm petri dishes and transfected the following day with 50 nM miR-146a, miCURY miR-146a inhibitor or Ctrl using Lipofectamine 2000 (Invitrogen). The following day the cell were transferred to 24-well plates where the cells were fixed at indicated time points in 4% paraformaldehyde, stained in a 0.1% crystal violet solution, and resuspended in 10% acetic acid. Sample absorbance was measured at 620 nm, and normalized to that of siGlo control transfected cells. Mean ± S.D. n = 4 /day. **Figure S5.** Expression of the mammalian signalosome 8 subunits (GPS1 and COPS2-8) in SNU638 cells transfected with miR-146a. Alteration in the expression of one subunit has been reported to affect the expression of the others. Using qPCR the expression of the subunits (GPS1 and COPS2-8) was therefore examined in SNU638 cells transfected with miR-146a. Only COPS8 mRNA is a direct target of miR-146a, indicated by open bars. The absence of miR-146a target sites is indicated by closed bars. The expression of COPS8 and COPS2 was reduced following miR-146a transfection. Mean ± S.D. * = p < 0.05, n = 4. COPS8 mRNA is directly targeted by miR-146a, while we assume that the expression of COPS2 is indirectly affected by miR-146a since the mRNA does not contain a miR-146a target site. **Figure S6.** Up-regulation of miR-146a in response to cancer-related cytokines. SNU638 cells were grown in media with 1% FCS overnight and subsequently stimulated with either LPA (10 μM), IL-1β (10 ng/ml) or vehicle for 6 h. The expression of miR-146a was measured by qPCR and normalized to the expression of U44. miR-146a expression is shown relative to the average expression in the control. Data are the mean ± S.D. (n = 4). **Figure S7.** Monocyte migration Boyden chamber assay. Monocyte migration against conditioned medium from control- or miR-146a-transfected SNU638 cells that were left untreated or stimulated with 25 μM LPA was followed over 6 hours. Monocyte migration against medium from LPA-stimulated SNU638 cells was increased compared to migration against medium from untreated cells, while monocyte migration against medium from miR-146a-transfected, LPA-stimulated cells was decreased compared to medium from control-transfected, LPA-stimulated cells. Data are shown as mean ± S.D, n = 3, * = P < 0.05. Monocytes were isolated as described. Monocytes were seeded in the upper chambers of Boyden chamber assay (Becton Dickinson, Heidelberg, Germany), 2.5x104 cells/well were seeded in RPMI1640 medium containing 1%. The lower chambers contained conditioned medium from SNU638 cells that had been transfected with Ctrl, miR-146a, or miCURY-miR-146a inhibitor and the following day left untreated or treated with 25 μM LPA (Avanti Polar Lipids) for 6 hours (n = 3). After 120-minute incubation in 5% CO2 at 37°C, non-migrating cells were scraped from the upper surface of the filter using a cotton plug. Cells on the lower surface were fixed with methanol and stained with methylene blue. The number of cells on the lower surface of the filter was determined microscopically by counting 3 high-power (x400) fields of constant area per well. To normalize all experiments, values were expressed as the fraction of number of cells that migrated through in the unconditioned control. Mean ± S.D. **Table S1** mRNA qRT-PCR primers (TAG Copenhagen, Copenhagen, Denmark). **Table S2** 3’UTR luciferase primers. Restriction sites are indicated with lower case lettters (TAG Copenhagen). **Table S3** Mutagenesis primers. **Table 4** Transcripts with predicted 3’UTR miR-146a target sites that were significantly down-regulated upon miR-146a transfection. Click here for file
