# Supplementary material for: microRNA-146a inhibits G protein-coupled receptor-mediated activation of NF-κB by targeting CARD10 and COPS8 in gastric cancer
Source: Mol Cancer. 2012 Sep 20;11:71. doi: 10.1186/1476-4598-11-71 (PMC3515505; doi:10.1186/1476-4598-11-71)
Supplement: Additional file 1 — Figure S1. Expression of miR-146a in the gastrin KO mice and in human gastric cancer. In situ hybridization detection of miR-146a expression. (A and B) miR-146a expression was absent in WT fundic mouse tissue, while miR-146a-positive cells (blue nuclei) were detected in the metaplastic fundic tissue from gastrin KO mice. (C and D) miR-146a expression was absent in normal human gastric tissue, while human gastric adenocarcinoma cells stained for miR-146a (blue nuclei). Original magnification x10, Scale bar = 100 μm. (PDF 1343 kb). Figure S2. Survival of gastric cancer patients with low or high tumor miR-146a expression. Kaplan-Meier overall survival curves according to miR-146a level. Although patients whose tumors have high expression of miR-146a seemed to have better survival than those with low expression, it was not significant, p = 0,31 (Mantel-Cox Test). Low expression was defined as those with a relative miR-146a expression below the median expression and high expression had relative expression above median. Figure S3. Relative expression of miR-146a in cell lines. The expression of miR-146a in cells lines determined by qPCR. Gastric cancer cell lines are indicated by red bars. Relative miR-146a expression was determined using TaqMan® MicroRNA Assay hsa-miR-146a according to the manufacturer’s protocol (Applied Biosystems). miR-146a levels were normalized to hsa-miR-191 (Applied Biosystems), which served as an endogenous control. Figure S4. Normal growth of SNU638 cells transfected with miR-146a in cell lines. 2,5 106 SNU638 cells were seeded in 10 cm petri dishes and transfected the following day with 50 nM miR-146a, miCURY miR-146a inhibitor or Ctrl using Lipofectamine 2000 (Invitrogen). The following day the cell were transferred to 24-well plates where the cells were fixed at indicated time points in 4% paraformaldehyde, stained in a 0.1% crystal violet solution, and resuspended in 10% acetic acid. Sample absorbance was measured at 620 nm, and normalized t [file 1476-4598-11-71-S1.pdf]

Supplementary data

Figure S1. Expression of miR-146a in the gastrin KO mice and in human gastric cancer.

In situ hybridization detection of miR-146a expression. (*A* and *B*) miR-146a expression was absent in WT fundic mouse tissue, while miR-146a-positive cells (blue nuclei) were detected in the metaplastic fundic tissue from gastrin KO mice. (*C* and *D*) miR-146a expression was absent in normal human gastric tissue, while human gastric adenocarcinoma cells stained for miR-146a (blue nuclei). Original magnification x10, Scale bar = 100 µm.

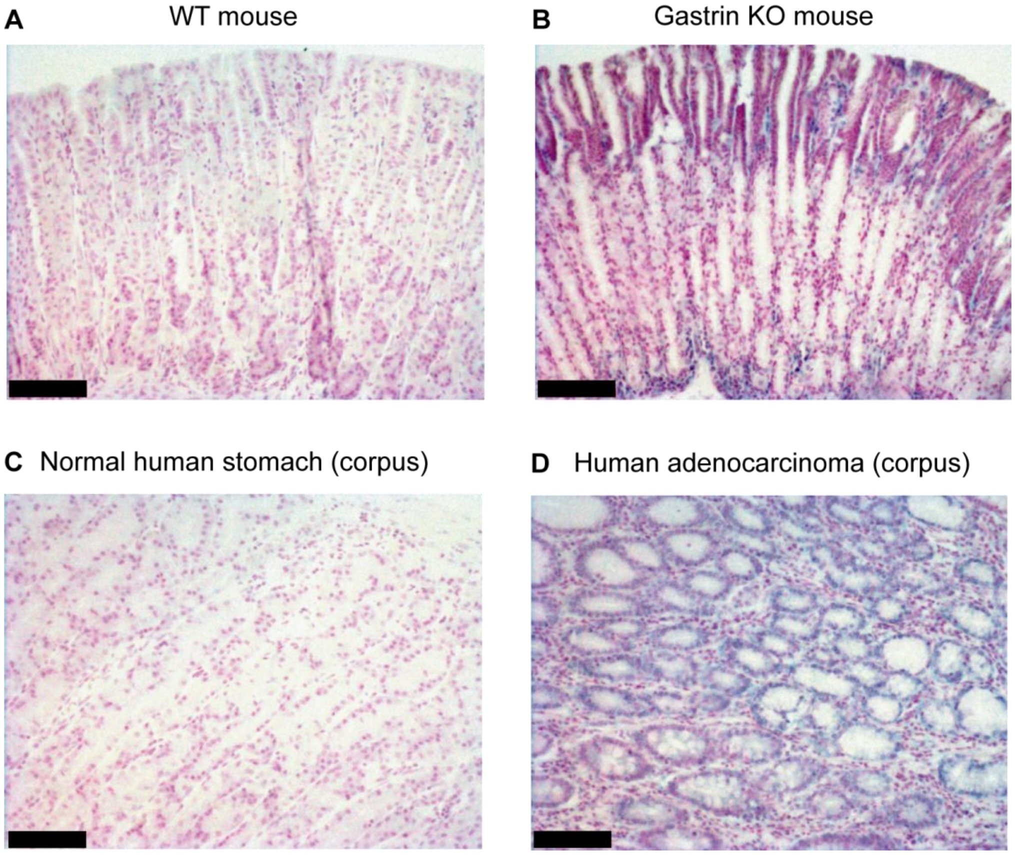

Figure S2. Survival of gastric cancer patients with low or high tumor miR-146a expression. Kaplan-Meier overall survival curves according to miR-146a level. Although patients whose tumors have high expression of miR-146a seemed to have better survival than those with low expression, it was not significant, p = 0,31 (Mantel-Cox Test). Low expression was defined as those with a relative miR-146a expression below the median expression and high expression had relative expression above median.

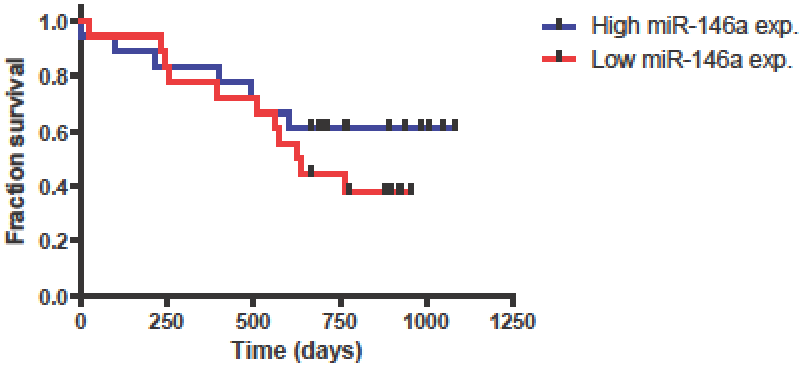

Figure S3. Relative expression of miR-146a in cell lines.

The expression of miR-146a in cells lines determined by qPCR. Gastric cancer cell lines are indicated by red bars. Relative miR-146a expression was determined using TaqMan® MicroRNA Assay hsa-miR-146a according to the manufacturer's protocol (Applied Biosystems). miR-146a levels were normalized to hsa-miR-191 (Applied Biosystems), which served as an endogenous control.

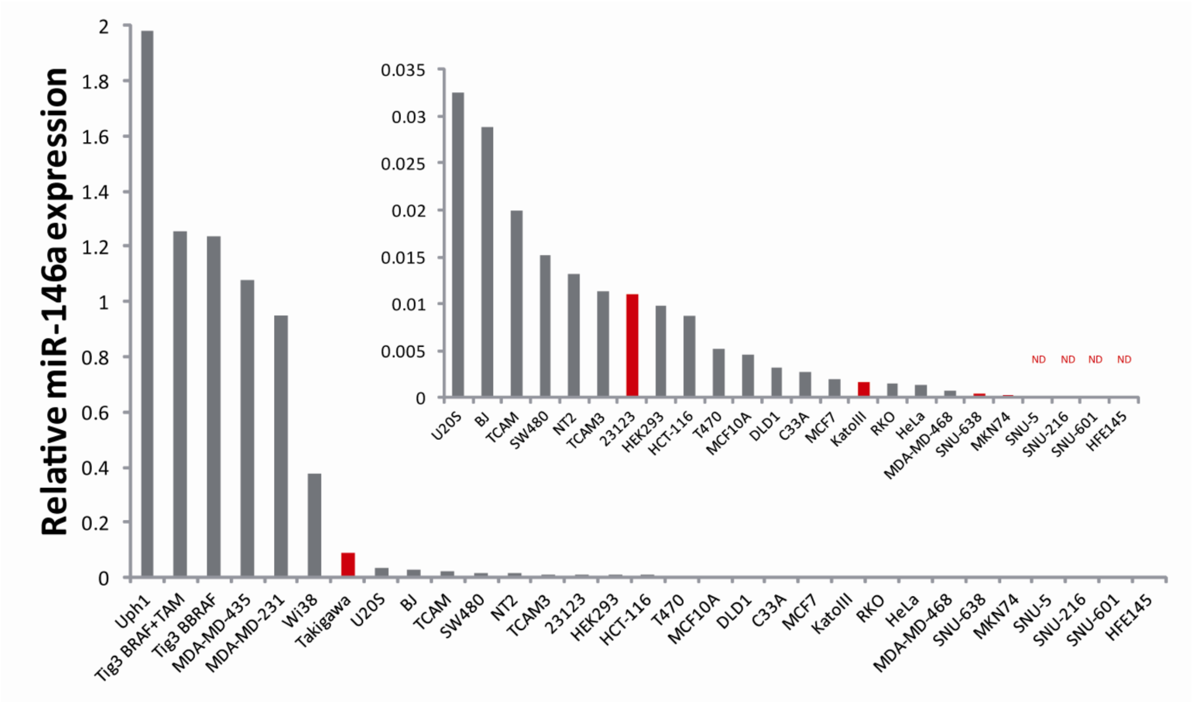

Figure S4. Normal growth of SNU638 cells transfected with miR-146a in cell lines.

2,5 10^6 SNU638 cells were seeded in 10 cm petri dishes and transfected the following day with 50 nM miR-146a, miCURY miR-146a inhibitor or Ctrl using Lipofectamine 2000 (Invitrogen). The following day the cell were transferred to 24-well plates where the cells were fixed at indicated time points in 4% paraformaldehyde, stained in a 0.1% crystal violet solution, and resuspended in 10% acetic acid. Sample absorbance was measured at 620 nm, and normalized to that of siGlo control transfected cells. Mean ± S.D. n = 4 /day.

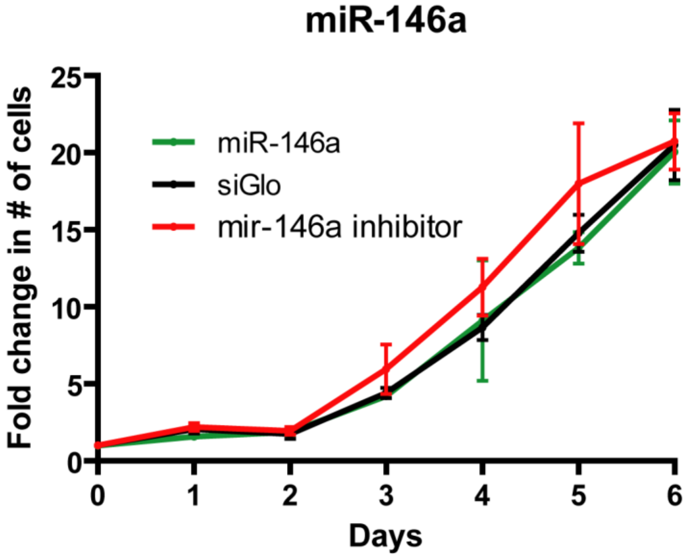

Figure S5 Expression of the mammalian signalosome 8 subunits (GPS1 and COPS2-8) in SNU638 cells transfected with miR-146a.

Alteration in the expression of one subunit has been reported to affect the expression of the others. Using qPCR the expression of the subunits (GPS1 and COPS2-8) was therefore examined in SNU638 cells transfected with miR-146a. Only COPS8 mRNA is a direct target of miR-146a, indicated by open bars. The absence of miR-146a target sites is indicated by closed bars. The expression of COPS8 and COPS2 was reduced following miR-146a transfection. Mean ± S.D. * = $p < 0.05$, n = 4. COPS8 mRNA is directly targeted by miR-146a, while we assume that the expression of COPS2 is indirectly affected by miR-146a since the mRNA does not contain a miR-146a target site.

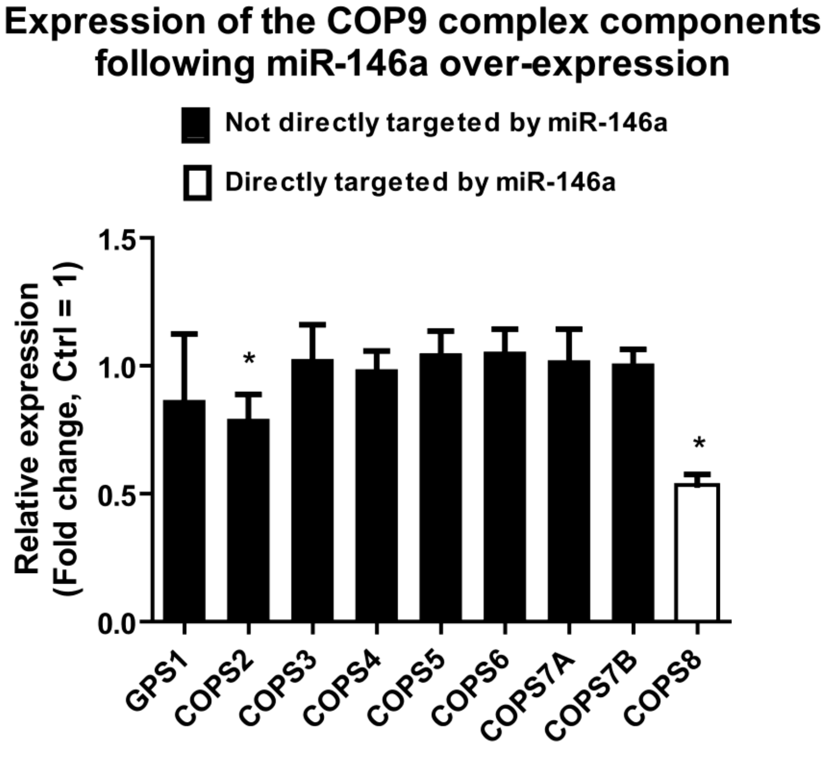

Figure S6. Up-regulation of miR-146a in response to cancer-related cytokines.

SNU638 cells were grown in media with 1% FCS overnight and subsequently stimulated with either LPA (10 µM), IL-1β (10 ng/ml) or vehicle for 6 h. The expression of miR-146a was measured by qPCR and normalized to the expression of U44. miR-146a expression is shown relative to the average expression in the control. Data are the mean ± S.D. (n = 4).

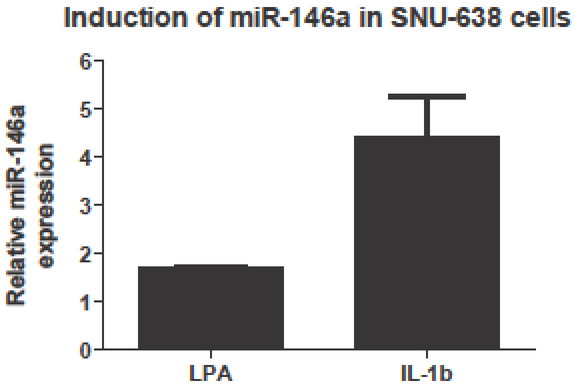

Figure S7 Monocyte migration Boyden chamber assay

Monocyte migration against conditioned medium from control- or miR-146a-transfected SNU638 cells that were left untreated or stimulated with 25 µM LPA was followed over 6 hours. Monocyte migration against medium from LPA-stimulated SNU638 cells was increased compared to migration against medium from untreated cells, while monocyte migration against medium from miR-146a-transfected, LPA-stimulated cells was decreased compared to medium from control-transfected, LPA-stimulated cells. Data are shown as mean ± S.D, n = 3, * = P < 0.05.

Monocytes were isolated as described. Monocytes were seeded in the upper chambers of Boyden chamber assay (Becton Dickinson, Heidelberg, Germany), $2.5x10^4$ cells/well were seeded in RPMI1640 medium containing 1%. The lower chambers contained conditioned medium from SNU638 cells that had been transfected with Ctrl, miR-146a, or miCURY-miR-146a inhibitor and the following day left untreated or treated with 25 µM LPA (Avanti Polar Lipids) for 6 hours (n=3). After 120-minute incubation in 5% CO2 at 37°C, non-migrating cells were scraped from the upper surface of the filter using a cotton plug. Cells on the lower surface were fixed with methanol and stained with methylene blue. The number of cells on the lower surface of the filter was determined microscopically by counting 3 high-power (x400) fields of constant area per well. To normalize all experiments, values were expressed as the fraction of number of cells that migrated through in the unconditioned control. Mean ± S.D.

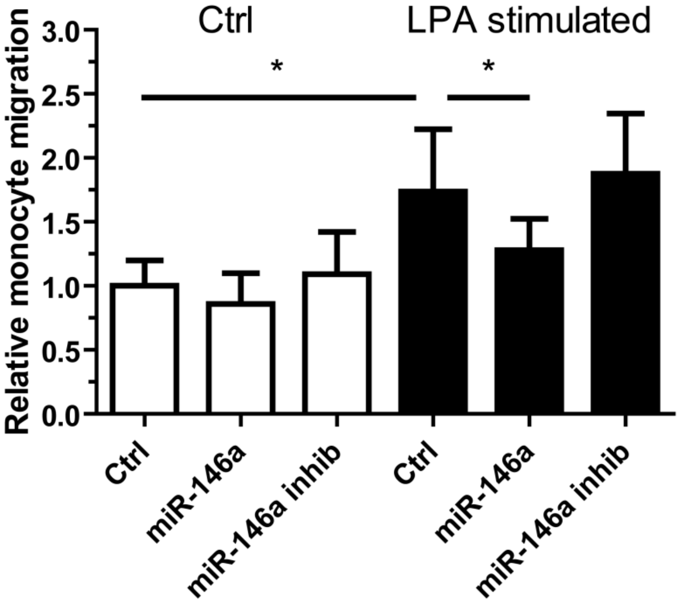

Ctrl
LPA stimulated
*
*
Relative monocyte migration
3.0
2.5
2.0
1.5
1.0
0.5
0.0
Ctrl
miR-146a
miR-146a inhib
Ctrl
miR-146a
miR-146a inhib

Table S1 **mRNA qRT-PCR primers (TAG Copenhagen, Copenhagen, Denmark)**

GAPDH FW	5’-CGACCACTTTGTCAAGCTCA-3’
GAPDH RV	5’-GGTGGTCCAGGGGTCTTACT-3’
CARD10 FW	5’-CACCGCACGCTGCAGAAGGA-3'
CARD10 RV	5’-CGGTCACGGCTCTGGATGGC-3'
COPS8 FW	5’-ACGCTTGATGTAATGGGCAGAATCA-3’
COPS8 RV	5’-AGCTGACCATACACTGGGGGTGT-3’
IRAK1 FW	5’-GGGCTGTGAAGACGCACGGT-3’
IRAK1 RV	5’- TGGGAGCAGCCCAGGCATCT-3’
IL6 FW	5'-AGATGTAGCCGCCCCACACA-3'
IL6 RV	5'-CTGCCAGTGCCTCTTTGCTGCTTT-3'
IL8 FW	5'-CTCTTGGCAGCCTTCCTGATT-3'
IL8 RV	5'-TATGCACTGACATCTAAGTTCTTTAGCA-3'
IL11 FW	5'-TCAGGGCACATGCCTCCCCT-3'
IL11 RV	5'-CTGGCCACAGGCTCAGCACG-3'
IL23A FW	5'-ATTTTCACAGGGGAGCCTTC-3'
IL23A RV	5'-GACTGAGGCTTGGAATCTGC-3'
CCL5 FW	5'-TACATTGCCCGCCCACTGCC-3'
CCL5 RV	5'-GGGTTGGCACACACTTGGCG-3'
CSF1 FW	5'-AGGGCAGCCCCCTGACTCAG-3'
CSF1 RV	5'-GGAGGATGGCCAGGGAGGGG-3'
PDGFB FW	5'-TGAATCGCTGCTGGGCGCTCTTC-3'
PDGFB RV	5'-AGGAGCGGATCGAGTGGTCACTC-3'

Table S2 3’UTR luciferase primers. Restriction sites are indicated with lower case lettters (TAG Copenhagen).

CARD10 3’UTR FW	5’-TATAacgcgtGGCCTCTCACCCCCAAGGCT-3’
CARD10 3’UTR RV	5’-TATAaagcttGCACGGTCCTGAGTGTGCCC-3’
COPS8 3’UTR FW	5’-TATAacgcgtAGCCTGCTCCAGTTCCCCCA-3’
COPS8 3’UTR RV	5’-TATAaagcttACGGCATGTGCTGGGACAGA-3’

Table S3 Mutagenesis primers

CARD10 Mut FW	5'-CAGGTTTGCACACTCAAGAAGACATGGGCAGGCTCAGGTC-3'
CARD10 Mut RV	5'-GACCTGAGCCTGCCCATGTCTTCTTGAGTGTGCAAACCTG-3’
COPS8 Mut FW	5'-CGTTATGCTGAATAGTTGTTGAAACAGAAGACATTTTGTAGTATTTAATAATCTGGATG-3'
COPS8 Mut RV	5'-CATCCAGATTATTAAATACTACAAAATGTCTTCTGTTTCAACAACTATTCAGCATAACG-3'

Table 4 Transcripts with predicted 3'UTR miR-146a target sites that were significantly down-regulated upon miR-146a transfection.

probeset	transcript	HGNC symbol	log2 fold change	p-value	3utr 6m	3utr 7m	3utr length
201587_s_at	ENST00000369980	IRAK1	-1.48	9.53E-04	2	2	1350
210026_s_at	ENST00000251973	CARD10	-1.04	8.60E-04	3	1	792
202141_s_at	ENST00000392008	COPS8	-1.01	2.75E-04	1	1	1102
227964_at	ENST00000317568	FRMD8	-0.98	5.52E-03	2	1	2159
218140_x_at	ENST00000273406	SRPRB	-0.92	3.21E-04	2	2	913
222768_s_at	ENST00000203001	TRMT6	-0.87	4.19E-03	2	0	695
202656_s_at	ENST00000313349	SERTAD2	-0.86	3.20E-02	4	1	4305
204928_s_at	ENST00000263512	SLC10A3	-0.84	1.81E-04	1	1	198
222447_at	ENST00000358154	METTL9	-0.84	5.13E-03	2	1	2038
225383_at	ENST00000370249	ZNF275	-0.82	1.43E-04	2	0	3418
225383_at	ENST00000346165	ZNF275	-0.82	1.43E-04	6	1	5153
204720_s_at	ENST00000263441	DNAJC6	-0.82	2.11E-03	3	0	2844
203497_at	ENST00000300651	MED1	-0.81	1.21E-02	3	2	3179
224436_s_at	ENST00000351220	NIPSNAP3A	-0.81	8.00E-03	1	0	786
1555673_at	ENST00000391591	ENSG00000212904	-0.81	4.10E-03	1	0	421
1555673_at	ENST00000391418	KRTAP2-3	-0.81	4.10E-03	1	0	424
203740_at	ENST00000258169	MPHOSPH6	-0.78	2.69E-03	1	1	569
202142_at	ENST00000354371	COPS8	-0.78	6.99E-03	1	1	1102
215780_s_at	ENST00000372692	SET	-0.77	1.01E-02	1	0	478
218536_at	ENST00000378386	MRS2	-0.77	2.44E-03	2	1	1887
229700_at	ENST00000343332	ZNF738	-0.76	4.45E-04	2	2	639
229700_at	ENST00000311015	ZNF738	-0.76	4.45E-04	3	2	1814
203342_at	ENST00000376582	TIMM17B	-0.76	1.53E-03	1	1	280
203342_at	ENST00000396779	TIMM17B	-0.76	1.53E-03	1	1	280
208990_s_at	ENST00000265866	HNRNPH3	-0.76	1.14E-02	1	0	1133
222728_s_at	ENST00000323981	TAF1D	-0.75	5.58E-03	1	1	1248
209094_at	ENST00000284031	DDAH1	-0.72	2.92E-03	2	0	2966
231984_at	ENST00000380172	MTAP	-0.71	9.72E-04	5	2	3954
209723_at	ENST00000380698	SERPINB9	-0.71	2.88E-02	1	0	2889
202349_at	ENST00000351698	TOR1A	-0.70	1.75E-04	1	1	1029
202071_at	ENST00000372733	SDC4	-0.70	4.16E-02	1	0	1973
209476_at	ENST00000267434	TXNDC1	-0.70	4.89E-03	1	1	1519
222653_at	ENST00000225573	PNPO	-0.70	2.65E-02	4	1	2526
221705_s_at	ENST00000369526	ENSG00000052723	-0.68	2.54E-02	1	1	773
221705_s_at	ENST00000369528	ENSG00000052723	-0.68	2.54E-02	1	1	4792
225658_at	ENST00000280098	SPOPL	-0.67	2.46E-03	2	1	4147
226843_s_at	ENST00000357464	PAPD5	-0.67	4.61E-02	2	0	5982
219361_s_at	ENST00000332810	AEN	-0.67	5.05E-03	2	1	1987
213287_s_at	ENST00000269576	KRT10	-0.66	5.57E-03	1	0	358
200825_s_at	ENST00000353883	HYOU1	-0.66	3.80E-03	2	1	1405
212440_at	ENST00000244227	SNRNP27	-0.65	2.72E-02	2	1	908

230398_at	ENST00000254051	TNS4	-0.65	9.93E-04	1	0	1758
213026_at	ENST00000274459	ATG12	-0.65	4.06E-02	2	1	1686
210231_x_at	ENST00000372688	SET	-0.63	1.60E-02	1	0	478
210231_x_at	ENST00000372686	SET	-0.63	1.60E-02	1	0	1724
200772_x_at	ENST00000341369	PTMAP4	-0.63	4.14E-02	1	0	687
208802_at	ENST00000342756	SRP72	-0.63	6.15E-03	1	1	1819
223135_s_at	ENST00000325805	BBX	-0.63	4.04E-02	1	0	393
212438_at	ENST00000244227	SNRNP27	-0.63	1.60E-02	2	1	908
201542_at	ENST00000373242	SAR1A	-0.63	2.12E-03	1	1	2272
207023_x_at	ENST00000269576	KRT10	-0.62	1.58E-03	1	0	358
218845_at	ENST00000344450	DUSP22	-0.62	1.14E-02	2	0	485
224796_at	ENST00000357668	ASAP1	-0.62	1.02E-02	1	1	2624
211998_at	ENST00000254810	H3F3B	-0.60	5.11E-03	1	0	2159
208097_s_at	ENST00000267434	TXNDC1	-0.60	2.20E-02	1	1	1519
213518_at	ENST00000295797	PRKCI	-0.60	1.98E-02	1	1	2855
223178_s_at	ENST00000319550	NT5DC1	-0.60	1.71E-03	2	0	1665
218150_at	ENST00000295087	ARL5A	-0.60	2.13E-03	2	0	2310
200999_s_at	ENST00000378026	CKAP4	-0.59	3.88E-02	2	1	999
208095_s_at	ENST00000342756	SRP72	-0.59	4.27E-02	1	1	1819
200630_x_at	ENST00000372686	SET	-0.59	1.19E-02	1	0	1724
222557_at	ENST00000370053	ENSG00000197457	-0.58	9.94E-03	1	1	422
222557_at	ENST00000358145	STMN3	-0.58	9.94E-03	1	1	1612
208114_s_at	ENST00000368219	ISG20L2	-0.58	3.42E-02	1	0	746
203682_s_at	ENST00000249760	IVD	-0.58	4.81E-02	1	1	601
212766_s_at	ENST00000368219	ISG20L2	-0.58	6.01E-03	1	0	746
228026_at	ENST00000369528	ENSG00000052723	-0.58	9.85E-03	1	1	4792
220987_s_at	ENST00000367157	NUAK2	-0.58	8.28E-03	2	0	1385
210633_x_at	ENST00000269576	KRT10	-0.57	8.02E-03	1	0	358
221568_s_at	ENST00000278193	LIN7C	-0.57	4.87E-03	1	1	4072
200777_s_at	ENST00000353957	BZW1	-0.57	1.28E-03	2	1	1658
201609_x_at	ENST00000343813	ICMT	-0.57	5.17E-03	1	0	3887
202933_s_at	ENST00000359834	YES1	-0.56	2.38E-02	1	1	2836
225471_s_at	ENST00000358335	AKT2	-0.56	2.34E-02	1	1	1136
225471_s_at	ENST00000392038	AKT2	-0.56	2.34E-02	1	1	3555
225471_s_at	ENST00000311278	AKT2	-0.56	2.34E-02	1	1	3555
214260_at	ENST00000392008	COPS8	-0.55	7.53E-03	1	1	1102
214260_at	ENST00000354371	COPS8	-0.55	7.53E-03	1	1	1102
224806_at	ENST00000316881	ENSG00000121060	-0.55	4.16E-02	1	0	3791
201707_at	ENST00000368072	PEX19	-0.55	2.77E-02	3	1	2734
222523_at	ENST00000296257	SENP2	-0.55	5.06E-03	1	0	1251
216384_x_at	ENST00000341369	PTMAP4	-0.54	4.77E-02	1	0	687
222627_at	ENST00000354504	VPS54	-0.54	6.44E-03	1	1	897
227696_at	ENST00000288063	EXOSC6	-0.54	2.95E-03	3	1	4304
206023_at	ENST00000264218	NMU	-0.54	3.48E-03	1	0	169
208549_x_at	ENST00000341369	PTMAP4	-0.53	3.56E-02	1	0	687
238121_at	ENST00000392993	GK5	-0.53	2.57E-04	1	1	8095

200740_s_at	ENST00000332859	SUMO2	-0.53	1.79E-02	1	0	1334
218511_s_at	ENST00000225573	PNPO	-0.53	2.05E-03	4	1	2526
201726_at	ENST00000351593	ELAVL1	-0.53	4.31E-02	1	1	1208
218624_s_at	ENST00000312426	ENSG00000175487	-0.52	2.20E-02	1	0	147
217815_at	ENST00000216297	SUPT16H	-0.52	3.11E-02	2	0	1194
224993_at	ENST00000252674	MLLT1	-0.52	8.92E-03	2	2	2660
211921_x_at	ENST00000341369	PTMAP4	-0.52	4.68E-02	1	0	687
213034_at	ENST00000375288	ENSG00000160584	-0.52	4.05E-03	1	0	2239
213034_at	ENST00000292055	ENSG00000160584	-0.52	4.05E-03	1	0	2239
213034_at	ENST00000375300	ENSG00000160584	-0.52	4.05E-03	1	0	2241
201195_s_at	ENST00000261622	SLC7A5P1	-0.51	4.99E-02	1	0	2946
226360_at	ENST00000332811	ZNRF3	-0.51	6.70E-04	4	1	3865
212621_at	ENST00000300128	TMEM194A	-0.51	2.03E-02	3	1	4231
212621_at	ENST00000379391	TMEM194A	-0.51	2.03E-02	3	1	4231
225309_at	ENST00000216252	PHF5A	-0.51	3.11E-02	2	0	680
212532_s_at	ENST00000293406	LSM12	-0.51	3.62E-02	1	0	1572
217718_s_at	ENST00000353703	YWHAB	-0.51	1.67E-02	1	1	2081
218122_s_at	ENST00000296257	SENP2	-0.51	6.17E-04	1	0	1251
215088_s_at	ENST00000342751	SDHC	-0.51	3.27E-02	2	1	2200
215088_s_at	ENST00000367975	SDHC	-0.51	3.27E-02	3	2	2307
200631_s_at	ENST00000372686	SET	-0.51	3.42E-02	1	0	1724
206858_s_at	ENST00000394331	HOXC6	-0.50	4.57E-02	2	0	861
226276_at	ENST00000308489	TMEM167A	-0.50	3.95E-02	1	0	917
223275_at	ENST00000370078	PRMT6	-0.50	2.35E-02	1	0	1451
226926_at	ENST00000379073	DMKN	-0.49	2.00E-02	1	1	692
202332_at	ENST00000396832	CSNK1E	-0.49	1.83E-02	1	0	1279
209001_s_at	ENST00000354910	ANAPC13	-0.49	2.55E-02	2	0	882
209076_s_at	ENST00000269395	WDR45L	-0.49	6.47E-03	1	1	1342
209076_s_at	ENST00000392325	WDR45L	-0.49	6.47E-03	1	1	1342
202657_s_at	ENST00000313349	SERTAD2	-0.48	3.81E-02	4	1	4305
226617_at	ENST00000295087	ARL5A	-0.48	3.71E-02	2	0	2310
223350_x_at	ENST00000278193	LIN7C	-0.48	8.97E-03	1	1	4072
227196_at	ENST00000254260	RHPN2	-0.48	1.59E-02	1	0	1403
221582_at	ENST00000366695	HIST3H2A	-0.48	1.65E-02	1	1	446
212872_s_at	ENST00000394251	MED20	-0.48	1.44E-03	3	1	1718
218187_s_at	ENST00000331434	C8orf33	-0.48	2.17E-03	1	0	1872
205128_x_at	ENST00000362012	PTGS1	-0.48	2.70E-04	2	0	3158
205128_x_at	ENST00000223423	PTGS1	-0.48	2.70E-04	2	0	3158
205128_x_at	ENST00000373698	PTGS1	-0.48	2.70E-04	2	0	1771
212434_at	ENST00000264954	GRPEL1	-0.47	5.00E-03	1	1	807
223243_s_at	ENST00000318130	EDEM3	-0.47	4.26E-02	2	1	3560
208804_s_at	ENST00000244020	SFRS6	-0.47	2.20E-03	3	3	2538
201694_s_at	ENST00000239938	EGR1	-0.47	1.41E-02	2	0	1234
226032_at	ENST00000350623	CASP2	-0.47	6.29E-03	2	1	2620
226032_at	ENST00000310447	CASP2	-0.47	6.29E-03	2	1	2620
201661_s_at	ENST00000392065	ENSG00000123983	-0.46	4.44E-02	1	0	2985

204285_s_at	ENST00000269518	PMAIP1	-0.46	1.28E-02	1	1	1457
204285_s_at	ENST00000316660	PMAIP1	-0.46	1.28E-02	1	1	1553
213793_s_at	ENST00000334082	HOMER1	-0.46	7.79E-04	1	0	2045
213793_s_at	ENST00000282260	HOMER1	-0.46	7.79E-04	1	0	2045
213793_s_at	ENST00000399166	HOMER1	-0.46	7.79E-04	3	0	3373
221493_at	ENST00000368608	TSPYL1	-0.46	4.27E-02	1	1	1939
231849_at	ENST00000394815	KRT80	-0.46	2.19E-02	3	2	2402
231849_at	ENST00000301446	KRT80	-0.46	2.19E-02	3	2	2402
231849_at	ENST00000313234	KRT80	-0.46	2.19E-02	3	2	2527
224232_s_at	ENST00000303204	PRELID1	-0.46	6.51E-03	1	0	378
211754_s_at	ENST00000263255	SLC25A17	-0.46	6.27E-03	1	0	1197
219487_at	ENST00000393262	BBS10	-0.45	9.19E-03	1	1	1327
219487_at	ENST00000313898	BBS10	-0.45	9.19E-03	1	1	1327
213852_at	ENST00000330165	RBM8A	-0.45	6.62E-03	1	0	2233
200702_s_at	ENST00000330836	DDX24	-0.45	1.49E-02	1	0	253
202143_s_at	ENST00000392008	COPS8	-0.45	5.96E-03	1	1	1102
202143_s_at	ENST00000354371	COPS8	-0.45	5.96E-03	1	1	1102
200776_s_at	ENST00000353957	BZW1	-0.44	4.59E-03	2	1	1658
202738_s_at	ENST00000299167	PHKB	-0.44	4.65E-03	1	1	374
202738_s_at	ENST00000323584	PHKB	-0.44	4.65E-03	2	1	879
200073_s_at	ENST00000313899	HNRNPD	-0.44	2.55E-02	1	1	871
200073_s_at	ENST00000352301	HNRNPD	-0.44	2.55E-02	1	1	871
200073_s_at	ENST00000353341	HNRNPD	-0.44	2.55E-02	1	1	871
200073_s_at	ENST00000307213	HNRNPD	-0.44	2.55E-02	1	1	351
212118_at	ENST00000266119	ENSG00000112448	-0.43	3.08E-02	3	0	1068
212118_at	ENST00000342248	ENSG00000112448	-0.43	3.08E-02	3	0	1253
212118_at	ENST00000400720	ENSG00000215641	-0.43	3.08E-02	3	0	1068
212118_at	ENST00000400719	ENSG00000215641	-0.43	3.08E-02	3	0	1253
212118_at	ENST00000377199	TRIM27	-0.43	3.08E-02	3	0	1068
223155_at	ENST00000300605	HDHD2	-0.43	1.39E-02	1	0	1271
218152_at	ENST00000336216	HMG20A	-0.43	4.81E-02	2	1	2638
218152_at	ENST00000381714	HMG20A	-0.43	4.81E-02	2	1	2638
204587_at	ENST00000218197	SLC25A14	-0.43	1.33E-04	1	0	409
204587_at	ENST00000339231	SLC25A14	-0.43	1.33E-04	1	0	409
204587_at	ENST00000361980	SLC25A14	-0.43	1.33E-04	1	0	409
227477_at	ENST00000298585	ZMYND19	-0.43	2.11E-03	1	0	464
219399_at	ENST00000278193	LIN7C	-0.42	4.33E-03	1	1	4072
224320_s_at	ENST00000378886	MCM8	-0.42	1.98E-02	1	0	790
224320_s_at	ENST00000265187	MCM8	-0.42	1.98E-02	1	0	790
224320_s_at	ENST00000378896	MCM8	-0.42	1.98E-02	1	0	790
224320_s_at	ENST00000399350	MCM8	-0.42	1.98E-02	1	0	790
224320_s_at	ENST00000378883	MCM8	-0.42	1.98E-02	1	0	790
208112_x_at	ENST00000320631	EHD1	-0.42	1.75E-02	1	0	1597
208112_x_at	ENST00000359393	EHD1	-0.42	1.75E-02	1	0	1602
225498_at	ENST00000217402	CHMP4B	-0.42	3.89E-02	1	0	803
226215_s_at	ENST00000377069	FBXL10	-0.42	3.64E-02	1	1	1017

226215_s_at	ENST00000377071	FBXL10	-0.42	3.64E-02	1	1	1189
226215_s_at	ENST00000397480	FBXL10	-0.42	3.64E-02	1	1	1189
226215_s_at	ENST00000397478	FBXL10	-0.42	3.64E-02	1	1	1299
225997_at	ENST00000309395	MOBKL1A	-0.42	2.56E-02	4	2	6117
205398_s_at	ENST00000327367	SMAD3	-0.42	3.46E-02	1	0	4658
40189_at	ENST00000372686	SET	-0.42	4.68E-02	1	0	1724
208837_at	ENST00000299705	TMED3	-0.41	2.13E-02	1	0	626
225281_at	ENST00000314400	C3orf17	-0.41	2.49E-02	2	1	3092
225281_at	ENST00000383675	C3orf17	-0.41	2.49E-02	2	1	3092
225281_at	ENST00000393857	C3orf17	-0.41	2.49E-02	2	1	3092
209039_x_at	ENST00000320631	EHD1	-0.41	2.90E-02	1	0	1597
209039_x_at	ENST00000359393	EHD1	-0.41	2.90E-02	1	0	1602
209037_s_at	ENST00000320631	EHD1	-0.41	7.06E-03	1	0	1597
209037_s_at	ENST00000359393	EHD1	-0.41	7.06E-03	1	0	1602
224630_at	ENST00000378239	C2orf30	-0.41	4.53E-02	1	1	180
224630_at	ENST00000185150	C2orf30	-0.41	4.53E-02	1	1	827
235177_at	ENST00000272839	FAM119A	-0.41	4.11E-03	1	0	929
217752_s_at	ENST00000324262	CNDP2	-0.40	1.22E-02	1	1	1053
217752_s_at	ENST00000324301	CNDP2	-0.40	1.22E-02	1	1	1053
243013_at	ENST00000393909	MRPL19	-0.40	2.60E-02	3	0	6923
222762_x_at	ENST00000273317	LIMD1	-0.40	4.74E-02	1	1	4204
213532_at	ENST00000310823	ADAM17	-0.40	8.70E-03	1	1	893
201464_x_at	ENST00000371222	JUN	-0.40	2.99E-02	1	0	1287
214113_s_at	ENST00000330165	RBM8A	-0.40	7.04E-03	1	0	2233
227862_at	ENST00000374108	C1orf225	-0.39	9.68E-03	1	0	1182
202337_at	ENST00000368279	BGLAP	-0.39	1.38E-02	2	0	453
202337_at	ENST00000368273	BGLAP	-0.39	1.38E-02	2	0	434
202337_at	ENST00000368277	BGLAP	-0.39	1.38E-02	2	0	441
209059_s_at	ENST00000371649	EDF1	-0.39	4.54E-02	1	0	337
209059_s_at	ENST00000224073	EDF1	-0.39	4.54E-02	1	0	165
227016_at	ENST00000262109	ERICH1	-0.39	3.17E-02	1	1	402
202716_at	ENST00000371621	PTPN1	-0.39	1.24E-03	1	1	1836
220172_at	ENST00000339506	C2orf37	-0.39	1.27E-02	4	1	3936
220172_at	ENST00000375255	C2orf37	-0.39	1.27E-02	4	1	3936
206555_s_at	ENST00000381337	THUMPD1	-0.39	4.96E-02	2	0	2715
206555_s_at	ENST00000396083	THUMPD1	-0.39	4.96E-02	3	0	3211
225961_at	ENST00000381271	KLHDC5	-0.39	5.83E-03	2	0	4873
202348_s_at	ENST00000351698	TOR1A	-0.39	1.26E-02	1	1	1029
209674_at	ENST00000008527	CRY1	-0.39	2.81E-02	1	1	635
209674_at	ENST00000319645	CRY1	-0.39	2.81E-02	1	1	635
236655_at	ENST00000379097	TPD52	-0.38	4.58E-03	2	1	3244
236655_at	ENST00000379096	TPD52	-0.38	4.58E-03	2	1	3244
222499_at	ENST00000372945	MRPS16	-0.38	4.34E-02	1	0	2007
202647_s_at	ENST00000369535	NRAS	-0.38	2.01E-02	1	1	1130
225160_x_at	ENST00000296932	GPR126	-0.38	6.38E-03	1	1	2866
202149_at	ENST00000379446	NEDD9	-0.38	2.94E-02	1	0	1861

202149_at	ENST00000339240	NEDD9	-0.38	2.94E-02	1	0	1861
202149_at	ENST00000397373	NEDD9	-0.38	2.94E-02	1	0	3199
201190_s_at	ENST00000313486	PITPNA	-0.38	3.36E-02	1	0	2568
226727_at	ENST00000325718	MLLT6	-0.37	4.70E-03	3	1	4180
201870_at	ENST00000372813	TOMM34	-0.37	3.44E-02	1	0	978
201870_at	ENST00000372810	TOMM34	-0.37	3.44E-02	1	0	890
218642_s_at	ENST00000399611	ENSG00000215121	-0.37	1.57E-02	1	0	2424
205745_x_at	ENST00000310823	ADAM17	-0.37	2.45E-02	1	1	893
212432_at	ENST00000264954	GRPEL1	-0.37	1.28E-02	1	1	807
218069_at	ENST00000319285	ENSG00000179958	-0.37	5.20E-03	1	0	533
226394_at	ENST00000358935	MARCH5	-0.37	2.16E-02	1	1	2757
221481_x_at	ENST00000307213	HNRNPD	-0.37	1.06E-02	1	1	351
221481_x_at	ENST00000352301	HNRNPD	-0.37	1.06E-02	1	1	871
221481_x_at	ENST00000353341	HNRNPD	-0.37	1.06E-02	1	1	871
221481_x_at	ENST00000313899	HNRNPD	-0.37	1.06E-02	1	1	871
232071_at	ENST00000393909	MRPL19	-0.37	1.59E-02	3	0	6923
225382_at	ENST00000095634	ZNF275	-0.37	2.41E-03	2	0	3604
225382_at	ENST00000370249	ZNF275	-0.37	2.41E-03	2	0	3418
225382_at	ENST00000346165	ZNF275	-0.37	2.41E-03	6	1	5153
1568763_s_at	ENST00000343123	ENSG00000188002	-0.37	2.81E-02	2	0	1023
60474_at	ENST00000399304	FERMT1	-0.37	3.12E-02	2	1	2431
60474_at	ENST00000217289	FERMT1	-0.37	3.12E-02	3	2	2327
210110_x_at	ENST00000265866	HNRNPH3	-0.37	5.77E-03	1	0	1133
200020_at	ENST00000315091	TARDBP	-0.36	3.21E-02	1	0	935
200020_at	ENST00000240185	TARDBP	-0.36	3.21E-02	1	0	2835
210588_x_at	ENST00000265866	HNRNPH3	-0.36	2.98E-02	1	0	1133
207127_s_at	ENST00000265866	HNRNPH3	-0.36	1.34E-02	1	0	1133
209632_at	ENST00000264977	PPP2R3A	-0.36	1.41E-03	3	0	2725
209632_at	ENST00000334546	PPP2R3A	-0.36	1.41E-03	3	0	2725
205322_s_at	ENST00000373036	MTF1	-0.36	4.51E-02	3	1	5568
1555784_s_at	ENST00000369980	IRAK1	-0.36	4.35E-02	2	2	1350
1555784_s_at	ENST00000393687	IRAK1	-0.36	4.35E-02	2	2	1350
1555784_s_at	ENST00000369974	IRAK1	-0.36	4.35E-02	2	2	1350
1555784_s_at	ENST00000393682	IRAK1	-0.36	4.35E-02	2	2	1350
213320_at	ENST00000330796	PRMT3	-0.36	3.13E-02	2	1	814
213320_at	ENST00000331079	PRMT3	-0.36	3.13E-02	2	1	814
227935_s_at	ENST00000371687	PCGF5	-0.36	1.82E-02	1	0	585
225634_at	ENST00000242351	ZC3HAV1	-0.36	3.44E-02	1	0	4072
200050_at	ENST00000301233	ZNF146	-0.36	4.93E-02	1	0	1445
200739_s_at	ENST00000397898	SUMO2	-0.35	1.53E-02	1	0	1288
200739_s_at	ENST00000332859	SUMO2	-0.35	1.53E-02	1	0	1334
47105_at	ENST00000358896	DUS2L	-0.35	1.08E-02	1	1	316
201573_s_at	ENST00000360541	ETF1	-0.35	2.79E-03	2	0	2206
208264_s_at	ENST00000261868	EIF3J	-0.35	3.68E-02	3	1	1654
217527_s_at	ENST00000320805	NFATC2IP	-0.35	4.19E-02	1	0	2581
213391_at	ENST00000342856	DPY19L4	-0.35	4.18E-02	2	0	1924

213391_at	ENST00000396156	DPY19L4	-0.35	4.18E-02	2	0	1924
213391_at	ENST00000396155	DPY19L4	-0.35	4.18E-02	2	0	1924
218617_at	ENST00000316891	TRIT1	-0.35	1.26E-02	2	1	708
218617_at	ENST00000046894	TRIT1	-0.35	1.26E-02	2	1	708
218617_at	ENST00000372825	TRIT1	-0.35	1.26E-02	2	1	708
218617_at	ENST00000372818	TRIT1	-0.35	1.26E-02	2	1	693
218617_at	ENST00000372823	TRIT1	-0.35	1.26E-02	2	1	708
229255_x_at	ENST00000393456	GOSR2	-0.35	5.24E-04	1	0	2607
213112_s_at	ENST00000389805	SQSTM1	-0.35	8.77E-03	2	2	1482
227447_at	ENST00000230640	SKIV2L2	-0.35	2.02E-02	1	0	806
211471_s_at	ENST00000263116	RAB36	-0.34	1.38E-02	1	0	2780
211471_s_at	ENST00000341989	RAB36	-0.34	1.38E-02	1	0	2780
221549_at	ENST00000253237	GRWD1	-0.34	4.74E-04	1	0	872
208093_s_at	ENST00000380025	NDEL1	-0.34	2.33E-03	1	1	1214
208093_s_at	ENST00000334527	NDEL1	-0.34	2.33E-03	1	1	1128
201535_at	ENST00000380680	UBL3	-0.34	2.89E-02	1	0	2846
223363_at	ENST00000288607	PSMG3	-0.34	1.31E-02	1	0	362
202306_at	ENST00000301788	POLR2G	-0.34	4.50E-02	1	0	202
222738_at	ENST00000281445	WWC2	-0.33	1.76E-03	3	2	5043
203364_s_at	ENST00000343175	KIAA0652	-0.33	6.43E-03	3	1	2180
203364_s_at	ENST00000312040	KIAA0652	-0.33	6.43E-03	3	1	2180
203364_s_at	ENST00000359513	KIAA0652	-0.33	6.43E-03	3	1	2180
211855_s_at	ENST00000218197	SLC25A14	-0.33	3.66E-03	1	0	409
211855_s_at	ENST00000339231	SLC25A14	-0.33	3.66E-03	1	0	409
211855_s_at	ENST00000361980	SLC25A14	-0.33	3.66E-03	1	0	409
227968_at	ENST00000319863	PDDC1	-0.33	4.59E-02	3	2	3672
227968_at	ENST00000354286	PDDC1	-0.33	4.59E-02	3	2	3672
220333_at	ENST00000340965	PAQR5	-0.33	2.75E-02	3	1	3819
220333_at	ENST00000395407	PAQR5	-0.33	2.75E-02	3	1	3825
203420_at	ENST00000259963	FAM8A1	-0.33	9.50E-03	1	0	3379
209330_s_at	ENST00000352301	HNRNPD	-0.33	4.13E-03	1	1	871
209330_s_at	ENST00000313899	HNRNPD	-0.33	4.13E-03	1	1	871
215813_s_at	ENST00000362012	PTGS1	-0.33	1.15E-03	2	0	3158
215813_s_at	ENST00000223423	PTGS1	-0.33	1.15E-03	2	0	3158
215813_s_at	ENST00000373698	PTGS1	-0.33	1.15E-03	2	0	1771
229129_at	ENST00000313899	HNRNPD	-0.33	2.18E-03	1	1	871
229129_at	ENST00000352301	HNRNPD	-0.33	2.18E-03	1	1	871
229129_at	ENST00000353341	HNRNPD	-0.33	2.18E-03	1	1	871
222703_s_at	ENST00000373044	YRDC	-0.32	2.75E-02	1	0	983
226284_at	ENST00000325144	ZBTB2	-0.32	3.24E-02	3	2	1405
212280_x_at	ENST00000400772	ENSG00000215697	-0.32	3.76E-02	1	0	1642
212280_x_at	ENST00000337606	ATG4B	-0.32	3.76E-02	1	0	1649
224657_at	ENST00000377482	ERRFI1	-0.32	7.56E-03	2	0	1475
233842_x_at	ENST00000023939	C20orf43	-0.32	1.66E-02	1	0	617
233842_x_at	ENST00000395881	C20orf43	-0.32	1.66E-02	1	0	795
233842_x_at	ENST00000357348	C20orf43	-0.32	1.66E-02	1	0	583

225111_s_at	ENST00000377026	NAPB	-0.32	7.23E-03	2	0	2847
225111_s_at	ENST00000398425	NAPB	-0.32	7.23E-03	2	0	2847
221480_at	ENST00000307213	HNRNPD	-0.32	5.00E-03	1	1	351
221480_at	ENST00000352301	HNRNPD	-0.32	5.00E-03	1	1	871
221480_at	ENST00000353341	HNRNPD	-0.32	5.00E-03	1	1	871
221480_at	ENST00000313899	HNRNPD	-0.32	5.00E-03	1	1	871
226463_at	ENST00000395862	ATP6V1C1	-0.32	4.64E-02	2	0	4310
225766_s_at	ENST00000337273	TNPO1	-0.32	4.39E-02	1	1	8060
236012_at	ENST00000333082	PSMF1	-0.32	1.44E-04	2	1	2702
236012_at	ENST00000335877	PSMF1	-0.32	1.44E-04	3	2	3298
200947_s_at	ENST00000277865	GLUD1	-0.32	3.07E-02	1	0	1261
200947_s_at	ENST00000394415	GLUD1	-0.32	3.07E-02	1	0	1261
1560648_s_at	ENST00000368608	TSPYL1	-0.32	8.55E-03	1	1	1939
210378_s_at	ENST00000322310	SSNA1	-0.32	3.32E-04	1	0	449
214210_at	ENST00000263255	SLC25A17	-0.32	1.26E-03	1	0	1197
227617_at	ENST00000340305	TMEM201	-0.32	2.18E-03	2	0	2657
242697_at	ENST00000316433	ENSG00000171817	-0.32	1.84E-02	1	1	830
242697_at	ENST00000343599	ENSG00000171817	-0.32	1.84E-02	1	1	830
223297_at	ENST00000272647	AMMECR1L	-0.31	2.23E-02	1	1	3464
223297_at	ENST00000393001	AMMECR1L	-0.31	2.23E-02	1	1	3457
224890_s_at	ENST00000341942	C7orf59	-0.31	2.20E-02	1	0	246
244743_x_at	ENST00000359735	ZNF138	-0.31	6.26E-03	2	2	1125
212451_at	ENST00000261847	ENSG00000138593	-0.31	3.45E-02	2	0	3474
226180_at	ENST00000323652	WDR36	-0.31	4.06E-02	2	1	3619
202706_s_at	ENST00000232607	UMPS	-0.31	1.57E-02	1	1	697
223099_s_at	ENST00000285737	LONP2	-0.31	3.66E-02	1	0	1694
227605_at	ENST00000394701	SCYE1	-0.31	2.04E-02	1	0	1533
223907_s_at	ENST00000314787	ENSG00000104637	-0.31	1.47E-02	1	0	436
223907_s_at	ENST00000354846	ENSG00000104637	-0.31	1.47E-02	1	0	821
1552711_a_at	ENST00000332439	CYB5D1	-0.31	1.05E-02	4	1	2668
233873_x_at	ENST00000358107	PAPD1	-0.31	4.77E-03	1	1	779
233873_x_at	ENST00000263063	PAPD1	-0.31	4.77E-03	1	1	779
223707_at	ENST00000314138	RPL27A	-0.31	2.13E-03	1	1	4049
201329_s_at	ENST00000360938	ETS2	-0.31	4.93E-02	1	0	2066
201329_s_at	ENST00000360214	ETS2	-0.31	4.93E-02	1	0	2066
201483_s_at	ENST00000225504	SUPT4H1	-0.31	8.31E-03	1	0	1068
203495_at	ENST00000292524	LRRC14	-0.31	1.80E-02	1	0	3695
45828_at	ENST00000221943	ATP5SL	-0.31	1.65E-02	1	0	912
45828_at	ENST00000301183	ATP5SL	-0.31	1.65E-02	1	0	953
217737_x_at	ENST00000023939	C20orf43	-0.31	1.99E-02	1	0	617
217737_x_at	ENST00000395881	C20orf43	-0.31	1.99E-02	1	0	795
217737_x_at	ENST00000357348	C20orf43	-0.31	1.99E-02	1	0	583
229232_at	ENST00000323443	LRRC57	-0.31	2.00E-02	2	1	1559
229232_at	ENST00000397130	LRRC57	-0.31	2.00E-02	2	1	1555
211678_s_at	ENST00000244061	RNF114	-0.30	3.65E-02	1	0	1742
224848_at	ENST00000265734	CDK6	-0.30	8.20E-04	2	1	10215

225615_at	ENST00000355609	IFFO2	-0.30	9.46E-03	1	0	4280
225615_at	ENST00000304963	IFFO2	-0.30	9.46E-03	1	0	4280
1558044_s_at	ENST00000288063	EXOSC6	-0.30	1.88E-02	3	1	4304
227921_at	ENST00000391598	ENSG00000212911	-0.30	4.66E-02	3	1	3278
209633_at	ENST00000264977	PPP2R3A	-0.30	1.94E-03	3	0	2725
209633_at	ENST00000334546	PPP2R3A	-0.30	1.94E-03	3	0	2725
209232_s_at	ENST00000300087	DCTN5	-0.30	3.28E-02	2	1	2717
213302_at	ENST00000314666	PFAS	-0.30	4.63E-02	1	0	1221
225765_at	ENST00000337273	TNPO1	-0.30	3.55E-02	1	1	8060
219444_at	ENST00000218147	BCORL1	-0.30	3.52E-02	1	1	1462
219444_at	ENST00000303743	BCORL1	-0.30	3.52E-02	1	1	1462
219444_at	ENST00000359304	BCORL1	-0.30	3.52E-02	2	1	2293
202737_s_at	ENST00000252816	LSM4	-0.30	1.71E-02	1	0	554
231835_at	ENST00000378425	C1orf93	-0.29	2.15E-02	1	1	2036
231835_at	ENST00000378424	C1orf93	-0.29	2.15E-02	1	1	2049
231835_at	ENST00000378427	C1orf93	-0.29	2.15E-02	1	1	2049
226651_at	ENST00000334082	HOMER1	-0.29	1.48E-03	1	0	2045
226651_at	ENST00000282260	HOMER1	-0.29	1.48E-03	1	0	2045
226651_at	ENST00000399166	HOMER1	-0.29	1.48E-03	3	0	3373
212481_s_at	ENST00000300933	TPM4	-0.29	2.66E-03	1	0	1252
214358_at	ENST00000353139	ACACA	-0.29	2.02E-02	1	0	2318
218034_at	ENST00000223136	FIS1	-0.29	4.23E-02	1	0	192
219683_at	ENST00000240093	FZD3	-0.29	2.23E-03	1	0	1431
223898_at	ENST00000366503	ZNF670	-0.29	3.39E-03	1	0	664
219648_at	ENST00000263268	CARD8	-0.29	2.05E-02	2	0	2272
212063_at	ENST00000278385	CD44	-0.29	3.68E-02	1	0	3066
212063_at	ENST00000279452	CD44	-0.29	3.68E-02	1	0	3066
212063_at	ENST00000352818	CD44	-0.29	3.68E-02	1	0	3066
212063_at	ENST00000263398	CD44	-0.29	3.68E-02	1	0	3066
212063_at	ENST00000278386	CD44	-0.29	3.68E-02	1	0	3112
218038_at	ENST00000221943	ATP5SL	-0.29	4.81E-02	1	0	912
218038_at	ENST00000301183	ATP5SL	-0.29	4.81E-02	1	0	953
222426_at	ENST00000265960	MAPKAP1	-0.28	4.27E-02	1	0	1493
222426_at	ENST00000350766	MAPKAP1	-0.28	4.27E-02	1	0	1493
222426_at	ENST00000373511	MAPKAP1	-0.28	4.27E-02	1	0	1493
222426_at	ENST00000373498	MAPKAP1	-0.28	4.27E-02	1	0	1493
222426_at	ENST00000373505	MAPKAP1	-0.28	4.27E-02	1	0	1493
222426_at	ENST00000373503	MAPKAP1	-0.28	4.27E-02	1	0	1493
222426_at	ENST00000394063	MAPKAP1	-0.28	4.27E-02	1	0	1493
222426_at	ENST00000373497	MAPKAP1	-0.28	4.27E-02	1	0	2393
222221_x_at	ENST00000320631	EHD1	-0.28	4.77E-02	1	0	1597
222221_x_at	ENST00000359393	EHD1	-0.28	4.77E-02	1	0	1602
218602_s_at	ENST00000380496	FAM29A	-0.28	1.91E-02	1	1	1332
217819_at	ENST00000276530	GOLGA7	-0.28	3.38E-02	1	0	1406
217819_at	ENST00000357743	GOLGA7	-0.28	3.38E-02	1	0	1406
239135_at	ENST00000261660	ENSG00000103381	-0.28	2.26E-02	2	1	5086

239101_at	ENST00000262650	ITCH	-0.28	1.33E-02	4	2	3598
239101_at	ENST00000374864	ITCH	-0.28	1.33E-02	4	2	3598
218570_at	ENST00000395288	KBTBD4	-0.28	4.34E-02	1	0	732
218570_at	ENST00000357777	KBTBD4	-0.28	4.34E-02	1	0	732
218570_at	ENST00000359900	KBTBD4	-0.28	4.34E-02	1	0	1362
219736_at	ENST00000282369	TRIM36	-0.28	2.99E-03	1	1	1722
41386_i_at	ENST00000254846	JMJD3	-0.28	4.32E-02	1	0	1267
224738_x_at	ENST00000372871	RPL7L1	-0.28	3.85E-02	1	0	3432
203338_at	ENST00000337537	PPP2R5E	-0.28	2.99E-03	1	0	1372
203160_s_at	ENST00000373479	RNF8	-0.28	4.72E-02	1	1	483
203160_s_at	ENST00000229866	RNF8	-0.28	4.72E-02	2	2	3885
224613_s_at	ENST00000360864	DNAJC5	-0.28	3.77E-02	2	0	2473
226850_at	ENST00000272902	SUMF1	-0.28	4.13E-02	1	1	998
226850_at	ENST00000383843	SUMF1	-0.28	4.13E-02	1	1	998
239377_at	ENST00000312234	EIF1AD	-0.27	1.78E-03	1	0	2053
233255_s_at	ENST00000257336	BIVM	-0.27	6.56E-03	1	1	1666
235005_at	ENST00000319194	DIS3L	-0.27	9.54E-03	2	1	585
201076_at	ENST00000355257	NHP2L1	-0.27	1.22E-02	2	0	997
201076_at	ENST00000215956	NHP2L1	-0.27	1.22E-02	2	0	997
217961_at	ENST00000273158	SLC25A38	-0.27	1.64E-02	1	0	707
223160_s_at	ENST00000337238	ENSG00000100796	-0.27	3.60E-02	1	0	1148
217808_s_at	ENST00000265960	MAPKAP1	-0.27	4.93E-02	1	0	1493
217808_s_at	ENST00000350766	MAPKAP1	-0.27	4.93E-02	1	0	1493
217808_s_at	ENST00000373511	MAPKAP1	-0.27	4.93E-02	1	0	1493
217808_s_at	ENST00000373498	MAPKAP1	-0.27	4.93E-02	1	0	1493
217808_s_at	ENST00000373505	MAPKAP1	-0.27	4.93E-02	1	0	1493
217808_s_at	ENST00000373503	MAPKAP1	-0.27	4.93E-02	1	0	1493
217808_s_at	ENST00000394063	MAPKAP1	-0.27	4.93E-02	1	0	1493
217808_s_at	ENST00000373497	MAPKAP1	-0.27	4.93E-02	1	0	2393
227237_x_at	ENST00000378737	ATAD3B	-0.27	3.76E-02	1	0	384
227237_x_at	ENST00000308647	ATAD3B	-0.27	3.76E-02	1	0	384
227237_x_at	ENST00000360489	ATAD3B	-0.27	3.76E-02	1	0	384
227237_x_at	ENST00000378759	ATAD3C	-0.27	3.76E-02	1	0	612
227237_x_at	ENST00000339113	ATAD3C	-0.27	3.76E-02	1	0	612
227237_x_at	ENST00000378756	ATAD3C	-0.27	3.76E-02	1	0	613
227237_x_at	ENST00000378755	ATAD3C	-0.27	3.76E-02	1	0	612
227983_at	ENST00000280571	RILPL2	-0.27	1.59E-02	1	0	502
227737_at	ENST00000273406	SRPRB	-0.27	1.97E-03	2	2	913
227204_at	ENST00000353265	PARD6G	-0.27	1.13E-02	2	2	2531
222006_at	ENST00000302787	LETM1	-0.27	1.48E-02	1	0	1391
217997_at	ENST00000266671	PHLDA1	-0.27	4.12E-02	1	0	4672
218727_at	ENST00000219320	SLC38A7	-0.27	3.71E-02	1	1	991
201053_s_at	ENST00000333082	PSMF1	-0.27	2.57E-02	2	1	2702
201053_s_at	ENST00000335877	PSMF1	-0.27	2.57E-02	3	2	3298
201466_s_at	ENST00000371222	JUN	-0.27	2.55E-02	1	0	1287
223518_at	ENST00000377036	DFFA	-0.27	5.80E-04	2	1	2486

210819_x_at	ENST00000388841	DIO2	-0.26	7.57E-03	2	1	2610
203068_at	ENST00000377658	KLHL21	-0.26	2.49E-02	1	0	2640
203068_at	ENST00000377663	KLHL21	-0.26	2.49E-02	2	0	4641
228698_at	ENST00000304501	SOX7	-0.26	9.73E-03	1	0	1970
226518_at	ENST00000228495	KCTD10	-0.26	4.27E-02	3	0	2940
222584_at	ENST00000245564	MSTO1	-0.26	4.50E-02	1	1	693
218296_x_at	ENST00000245564	MSTO1	-0.26	1.56E-02	1	1	693
225659_at	ENST00000280098	SPOPL	-0.26	2.79E-02	2	1	4147
202515_at	ENST00000346964	DLG1	-0.26	3.60E-02	1	1	2063
202515_at	ENST00000314062	DLG1	-0.26	3.60E-02	1	1	2063
202515_at	ENST00000381807	DLG1	-0.26	3.60E-02	1	1	2063
202515_at	ENST00000357674	DLG1	-0.26	3.60E-02	1	1	2063
202515_at	ENST00000359922	DLG1	-0.26	3.60E-02	1	1	2063
213206_at	ENST00000393456	GOSR2	-0.26	3.24E-02	1	0	2607
218867_s_at	ENST00000261318	C12orf49	-0.26	1.80E-02	2	0	2017
201513_at	ENST00000389682	TSN	-0.26	2.26E-03	1	0	2486
215954_s_at	ENST00000248420	C19orf29	-0.25	1.56E-02	1	0	339
218998_at	ENST00000322940	C9orf6	-0.25	5.10E-03	2	1	1264
218998_at	ENST00000374621	C9orf6	-0.25	5.10E-03	2	1	1264
203044_at	ENST00000254190	CHSY1	-0.25	1.49E-02	1	0	1660
221648_s_at	ENST00000375826	AGMAT	-0.25	2.11E-02	1	1	994
201577_at	ENST00000336097	NME1	-0.25	2.22E-02	1	1	244
201577_at	ENST00000311032	NME1	-0.25	2.22E-02	1	1	186
201577_at	ENST00000393196	NME1	-0.25	2.22E-02	1	1	186
203848_at	ENST00000269701	AKAP8	-0.25	3.32E-02	1	1	1385
219486_at	ENST00000358896	DUS2L	-0.25	4.48E-02	1	1	316
215631_s_at	ENST00000359957	BRMS1	-0.25	2.52E-02	1	0	549
207749_s_at	ENST00000264977	PPP2R3A	-0.25	3.29E-03	3	0	2725
207749_s_at	ENST00000334546	PPP2R3A	-0.25	3.29E-03	3	0	2725
202836_s_at	ENST00000269601	TXNL4A	-0.25	3.99E-02	1	0	816
228972_at	ENST00000313486	PITPNA	-0.25	2.14E-02	1	0	2568
226037_s_at	ENST00000400856	ENSG00000215760	-0.25	2.93E-02	2	0	1861
226037_s_at	ENST00000341864	TAF9B	-0.25	2.93E-02	2	0	1861
1554762_a_at	ENST00000281445	WWC2	-0.25	1.79E-02	3	2	5043
225756_at	ENST00000359867	CSNK1E	-0.25	2.77E-02	1	0	1279
225756_at	ENST00000396832	CSNK1E	-0.25	2.77E-02	1	0	1279
208840_s_at	ENST00000359707	G3BP2	-0.25	3.59E-02	2	0	2647
208840_s_at	ENST00000357854	G3BP2	-0.25	3.59E-02	2	0	2647
208840_s_at	ENST00000395719	G3BP2	-0.25	3.59E-02	2	0	2647
210059_s_at	ENST00000211287	MAPK13	-0.25	4.76E-02	1	0	692
210059_s_at	ENST00000373761	MAPK13	-0.25	4.76E-02	1	0	677
210059_s_at	ENST00000373770	MAPK13	-0.25	4.76E-02	1	0	677
210059_s_at	ENST00000373766	MAPK13	-0.25	4.76E-02	1	0	849
228517_at	ENST00000296214	C1orf149	-0.25	2.16E-02	1	0	785
228517_at	ENST00000373075	C1orf149	-0.25	2.16E-02	1	0	921
228517_at	ENST00000373074	C1orf149	-0.25	2.16E-02	1	0	788

218947_s_at	ENST00000358107	PAPD1	-0.24	3.81E-02	1	1	779
218947_s_at	ENST00000263063	PAPD1	-0.24	3.81E-02	1	1	779
204156_at	ENST00000375288	ENSG00000160584	-0.24	2.00E-03	1	0	2239
204156_at	ENST00000292055	ENSG00000160584	-0.24	2.00E-03	1	0	2239
204156_at	ENST00000375300	ENSG00000160584	-0.24	2.00E-03	1	0	2241
223398_at	ENST00000375464	C9orf89	-0.24	4.40E-02	1	0	176
212084_at	ENST00000272438	TEX261	-0.24	2.97E-02	2	1	2660
202095_s_at	ENST00000350051	BIRC5	-0.24	2.94E-02	2	0	2080
202095_s_at	ENST00000301633	BIRC5	-0.24	2.94E-02	2	0	2080
202095_s_at	ENST00000374948	BIRC5	-0.24	2.94E-02	2	0	1977
237159_x_at	ENST00000396654	AP1S3	-0.24	2.78E-03	1	0	3385
1552946_at	ENST00000315849	ZNF114	-0.24	1.82E-02	1	0	728
204085_s_at	ENST00000377453	CLN5	-0.24	1.39E-02	1	1	1547
232158_x_at	ENST00000295461	NPAL1	-0.24	1.27E-02	1	0	4001
213913_s_at	ENST00000229088	TBC1D30	-0.24	4.40E-02	1	1	2515
212931_at	ENST00000335626	TCF20	-0.23	4.05E-03	1	1	1328
212931_at	ENST00000359486	TCF20	-0.23	4.05E-03	1	1	1390
223680_at	ENST00000392143	ENSG00000198182	-0.23	4.02E-02	1	0	1665
223680_at	ENST00000355202	ENSG00000198182	-0.23	4.02E-02	1	0	1665
213278_at	ENST00000221086	MTMR9	-0.23	1.90E-03	4	0	5355
226109_at	ENST00000284881	C21orf91	-0.23	1.05E-02	2	0	4447
228363_at	ENST00000371199	XIAP	-0.23	3.28E-02	3	1	6791
228363_at	ENST00000355640	XIAP	-0.23	3.28E-02	3	1	6784
227627_at	ENST00000396609	C8orf44	-0.23	3.39E-02	2	2	2612
209270_at	ENST00000367030	LAMB3	-0.23	1.53E-02	1	1	294
209270_at	ENST00000391911	LAMB3	-0.23	1.53E-02	1	1	396
209270_at	ENST00000356082	LAMB3	-0.23	1.53E-02	1	1	396
229091_s_at	ENST00000265992	CCNJ	-0.23	3.01E-02	1	1	2624
222207_x_at	ENST00000320339	CXorf56	-0.23	4.91E-02	1	1	1578
224709_s_at	ENST00000360515	CDC42SE2	-0.23	3.32E-02	1	0	2787
224709_s_at	ENST00000395246	CDC42SE2	-0.23	3.32E-02	1	0	2787
225306_s_at	ENST00000359232	SLC25A29	-0.23	3.78E-02	1	0	1167
225306_s_at	ENST00000360662	SLC25A29	-0.23	3.78E-02	1	0	1167
225306_s_at	ENST00000392908	SLC25A29	-0.23	3.78E-02	1	0	1602
56821_at	ENST00000219320	SLC38A7	-0.23	3.15E-02	1	1	991
222547_at	ENST00000324219	MAP4K4	-0.22	5.10E-03	3	0	3440
222547_at	ENST00000350878	MAP4K4	-0.22	5.10E-03	3	0	3440
222547_at	ENST00000347699	MAP4K4	-0.22	5.10E-03	3	0	3440
222547_at	ENST00000350198	MAP4K4	-0.22	5.10E-03	3	0	3440
222547_at	ENST00000302217	MAP4K4	-0.22	5.10E-03	3	0	3440
226544_x_at	ENST00000244777	TXNDC5	-0.22	1.98E-03	1	0	1665
226544_x_at	ENST00000397457	TXNDC5	-0.22	1.98E-03	1	0	2082
218751_s_at	ENST00000281708	FBXW7	-0.22	1.35E-02	1	0	1623
218751_s_at	ENST00000393956	FBXW7	-0.22	1.35E-02	1	0	1622
218751_s_at	ENST00000296555	FBXW7	-0.22	1.35E-02	1	0	1623
218751_s_at	ENST00000263981	FBXW7	-0.22	1.35E-02	1	0	1622

212619_at	ENST00000300128	TMEM194A	-0.22	2.99E-02	3	1	4231
212619_at	ENST00000379391	TMEM194A	-0.22	2.99E-02	3	1	4231
1554410_a_at	ENST00000329959	WBSCR16	-0.22	1.51E-02	1	0	855
213296_at	ENST00000306256	RER1	-0.22	3.63E-02	1	0	2311
202852_s_at	ENST00000261880	ENSG00000103591	-0.22	3.16E-02	1	1	1148
218884_s_at	ENST00000281543	GUF1	-0.22	6.57E-03	1	0	1998
219470_x_at	ENST00000265992	CCNJ	-0.22	3.34E-03	1	1	2624
219092_s_at	ENST00000287996	IPPK	-0.22	2.14E-02	2	1	2648
201691_s_at	ENST00000379097	TPD52	-0.22	4.26E-02	2	1	3244
201691_s_at	ENST00000379096	TPD52	-0.22	4.26E-02	2	1	3244
242804_at	ENST00000382857	POLN	-0.21	3.22E-02	2	0	2628
222481_at	ENST00000254616	FXC1	-0.21	1.10E-03	1	1	2459
243176_at	ENST00000295087	ARL5A	-0.21	1.73E-02	2	0	2310
218060_s_at	ENST00000219281	C16orf57	-0.21	3.98E-02	1	0	1372
238454_at	ENST00000316433	ENSG00000171817	-0.21	1.64E-03	1	1	830
238454_at	ENST00000343599	ENSG00000171817	-0.21	1.64E-03	1	1	830
218421_at	ENST00000216264	CERK	-0.21	2.46E-02	1	1	2711
212852_s_at	ENST00000367446	TROVE2	-0.21	3.14E-02	1	0	1582
218647_s_at	ENST00000373044	YRDC	-0.21	3.62E-02	1	0	983
1555823_at	ENST00000325438	PACS2	-0.21	3.93E-02	1	1	3475
202616_s_at	ENST00000369957	MECP2	-0.21	1.38E-02	2	1	8554
202616_s_at	ENST00000303391	MECP2	-0.21	1.38E-02	2	1	8554
202616_s_at	ENST00000369964	MECP2	-0.21	1.38E-02	2	1	8794
202616_s_at	ENST00000393671	MECP2	-0.21	1.38E-02	2	1	8554
223682_s_at	ENST00000312234	EIF1AD	-0.21	8.48E-03	1	0	2053
221264_s_at	ENST00000240185	TARDBP	-0.21	3.14E-02	1	0	2835
226782_at	ENST00000310862	SLC25A30	-0.20	4.62E-02	1	0	2653
204877_s_at	ENST00000308893	TAOK2	-0.20	2.50E-02	1	0	418
219056_at	ENST00000336617	RNASEH2B	-0.20	2.29E-02	1	0	256
227893_at	ENST00000321517	C9orf130	-0.20	2.28E-02	1	0	836
1553423_a_at	ENST00000285013	SLFN13	-0.20	1.97E-02	3	1	5499
1553423_a_at	ENST00000360502	SLFN13	-0.20	1.97E-02	3	1	5499
226833_at	ENST00000332439	CYB5D1	-0.20	2.62E-02	4	1	2668
225678_at	ENST00000355209	POLR3H	-0.20	1.31E-03	2	1	3479
225678_at	ENST00000337566	POLR3H	-0.20	1.31E-03	2	1	3479
225678_at	ENST00000396504	POLR3H	-0.20	1.31E-03	2	1	3479
217789_at	ENST00000362031	SNX6	-0.20	4.25E-02	1	1	1710
217789_at	ENST00000396526	SNX6	-0.20	4.25E-02	1	1	2028
1555964_at	ENST00000336125	ARL17	-0.20	6.29E-04	5	3	3298
1555964_at	ENST00000398255	ARL17P1	-0.20	6.29E-04	5	3	3298
223208_at	ENST00000228495	KCTD10	-0.20	4.55E-02	3	0	2940
203307_at	ENST00000383450	ENSG00000206412	-0.20	4.06E-02	3	1	4695
203307_at	ENST00000383596	ENSG00000206492	-0.20	4.06E-02	3	1	4695
204541_at	ENST00000312932	SEC14L2	-0.20	1.60E-02	1	0	1432
213146_at	ENST00000254846	JMJD3	-0.20	1.58E-02	1	0	1267
230134_s_at	ENST00000373670	RC3H2	-0.20	2.30E-02	1	0	5071

244495_x_at	ENST00000383233	C18orf45	-0.20	1.62E-02	1	0	1994
244495_x_at	ENST00000256907	C18orf45	-0.20	1.62E-02	1	0	2356
231149_s_at	ENST00000301831	ULK4	-0.19	5.27E-03	1	0	327
222551_s_at	ENST00000331434	C8orf33	-0.19	1.82E-02	1	0	1872
41387_r_at	ENST00000254846	JMJD3	-0.19	3.36E-02	1	0	1267
217857_s_at	ENST00000330165	RBM8A	-0.19	5.67E-03	1	0	2233
209624_s_at	ENST00000340941	MCCC2	-0.19	2.08E-02	1	0	538
209624_s_at	ENST00000323375	MCCC2	-0.19	2.08E-02	1	0	538
204024_at	ENST00000297438	OSGIN2	-0.19	3.98E-02	1	1	2336
205207_at	ENST00000258743	IL6	-0.19	3.86E-02	1	0	415
223498_at	ENST00000395530	SPECC1	-0.19	3.00E-02	3	1	4961
225445_at	ENST00000288561	ENSG00000157741	-0.19	2.32E-02	3	0	10400
222561_at	ENST00000254770	LANCL2	-0.18	9.85E-03	1	0	2422
205340_at	ENST00000230122	ZBTB24	-0.18	3.90E-02	1	0	3335
221992_at	ENST00000359724	ENSG00000196877	-0.18	3.01E-02	1	0	808
218362_s_at	ENST00000377767	DIS3	-0.18	4.13E-02	4	0	4391
218362_s_at	ENST00000377780	DIS3	-0.18	4.13E-02	4	0	4391
227880_s_at	ENST00000316916	TMEM185A	-0.18	4.10E-02	2	0	1456
204148_s_at	ENST00000310842	POMZP3	-0.18	1.25E-02	1	0	237
228851_s_at	ENST00000354702	ENSA	-0.18	3.79E-02	1	0	2359
228851_s_at	ENST00000271690	ENSA	-0.18	3.79E-02	1	0	2359
228851_s_at	ENST00000356527	ENSA	-0.18	3.79E-02	1	0	2359
222689_at	ENST00000278544	PHCA	-0.18	3.38E-02	2	1	2591
223532_at	ENST00000393537	ANKRD39	-0.18	3.55E-02	1	1	260
224839_s_at	ENST00000340124	GPT2	-0.18	2.43E-02	3	0	2289
209164_s_at	ENST00000392975	CYB561	-0.18	3.44E-02	1	0	2095
209164_s_at	ENST00000392976	CYB561	-0.18	3.44E-02	1	0	2095
209164_s_at	ENST00000360793	CYB561	-0.18	3.44E-02	1	0	2095
239572_at	ENST00000241125	GJA3	-0.18	4.84E-02	1	0	3723
211784_s_at	ENST00000258962	SFRS1	-0.18	1.07E-02	2	0	4483
213466_at	ENST00000248139	RAB40C	-0.18	4.49E-02	2	0	1649
205995_x_at	ENST00000310864	IQCB1	-0.18	3.45E-02	1	0	346
205995_x_at	ENST00000349820	IQCB1	-0.18	3.45E-02	1	0	346
205995_x_at	ENST00000393650	IQCB1	-0.18	3.45E-02	1	0	1121
219576_at	ENST00000370661	MAP7D3	-0.17	4.55E-02	1	0	1824
226839_at	ENST00000331552	NR2C2AP	-0.17	1.99E-02	1	0	508
219615_s_at	ENST00000359534	KCNK5	-0.17	1.96E-03	3	0	1918
221002_s_at	ENST00000265450	TSPAN14	-0.17	1.51E-02	1	0	1652
221002_s_at	ENST00000341863	TSPAN14	-0.17	1.51E-02	1	0	1652
240437_at	ENST00000375890	CASP9	-0.17	3.54E-02	1	0	4662
233446_at	ENST00000262095	ONECUT2	-0.17	8.54E-03	8	3	14574
232118_at	ENST00000378868	CRLS1	-0.17	8.52E-03	1	0	3093
1555543_a_at	ENST00000356970	CLCC1	-0.17	2.32E-03	1	0	3039
1555543_a_at	ENST00000369968	CLCC1	-0.17	2.32E-03	1	0	3039
1555543_a_at	ENST00000369969	CLCC1	-0.17	2.32E-03	1	0	3039
1555543_a_at	ENST00000369970	CLCC1	-0.17	2.32E-03	1	0	3039

1555543_a_at	ENST00000369971	CLCC1	-0.17	2.32E-03	1	0	3039
215260_s_at	ENST00000395423	TCF3	-0.17	7.68E-03	1	0	2413
215260_s_at	ENST00000262965	TCF3	-0.17	7.68E-03	1	0	2413
215260_s_at	ENST00000344749	TCF3	-0.17	7.68E-03	1	0	2413
241727_x_at	ENST00000314636	DHFRL1	-0.16	2.35E-02	1	0	3024
241727_x_at	ENST00000394221	DHFRL1	-0.16	2.35E-02	1	0	1987
225042_s_at	ENST00000228515	FAM130A1	-0.16	2.74E-02	2	0	2539
223546_x_at	ENST00000337351	LUC7L	-0.16	3.27E-02	1	0	993
223546_x_at	ENST00000397783	LUC7L	-0.16	3.27E-02	1	1	402
1558426_x_at	ENST00000356387	ORAI2	-0.16	4.43E-02	1	0	1664
231249_at	ENST00000396885	ENSG00000198198	-0.16	3.60E-02	1	0	2153
231249_at	ENST00000372442	ENSG00000198198	-0.16	3.60E-02	1	0	2153
226714_at	ENST00000314471	SAMD4B	-0.16	1.12E-02	1	0	1368
212349_at	ENST00000375749	POFUT1	-0.16	1.62E-02	3	0	4000
222885_at	ENST00000296420	EMCN	-0.16	5.00E-02	1	0	584
222885_at	ENST00000305864	EMCN	-0.16	5.00E-02	1	0	584
234688_x_at	ENST00000380255	CNTROB	-0.16	7.87E-04	1	0	951
234688_x_at	ENST00000380262	CNTROB	-0.16	7.87E-04	1	0	66
234688_x_at	ENST00000303707	CNTROB	-0.16	7.87E-04	1	0	67
1565772_at	ENST00000356341	PAK1	-0.16	4.35E-02	1	0	1258
1565772_at	ENST00000278568	PAK1	-0.16	4.35E-02	1	0	1283
1555478_at	ENST00000335108	C17orf82	-0.16	2.54E-02	1	0	549
241367_at	ENST00000333437	ENSG00000182459	-0.16	3.22E-02	1	0	1124
32062_at	ENST00000292524	LRRC14	-0.16	2.20E-02	1	0	3695
203865_s_at	ENST00000389861	ADARB1	-0.16	4.34E-02	2	0	2393
203865_s_at	ENST00000389863	ADARB1	-0.16	4.34E-02	2	0	1005
203865_s_at	ENST00000348831	ADARB1	-0.16	4.34E-02	2	0	4362
203865_s_at	ENST00000389862	ADARB1	-0.16	4.34E-02	2	0	2393
203865_s_at	ENST00000389860	ADARB1	-0.16	4.34E-02	2	0	2393
203865_s_at	ENST00000360697	ADARB1	-0.16	4.34E-02	2	0	4363
220097_s_at	ENST00000335464	TMEM104	-0.16	2.51E-02	1	0	1792
1553995_a_at	ENST00000369651	NT5E	-0.15	1.28E-02	1	1	1774
1553995_a_at	ENST00000257770	NT5E	-0.15	1.28E-02	1	1	1774
223322_at	ENST00000304534	RASSF5	-0.15	4.91E-02	1	1	2305
223322_at	ENST00000367117	RASSF5	-0.15	4.91E-02	1	1	2435
223322_at	ENST00000355294	RASSF5	-0.15	4.91E-02	1	1	2305
223322_at	ENST00000338603	RASSF5	-0.15	4.91E-02	1	1	2245
206879_s_at	ENST00000394770	NRG2	-0.15	3.97E-02	1	0	1573
212585_at	ENST00000261183	OSBPL8	-0.15	4.37E-02	1	1	4080
212585_at	ENST00000393249	OSBPL8	-0.15	4.37E-02	1	1	4080
225128_at	ENST00000323468	KDELC2	-0.15	1.57E-02	1	1	2702
234734_s_at	ENST00000315183	TNRC6A	-0.15	2.72E-02	3	0	1068
234734_s_at	ENST00000395801	TNRC6A	-0.15	2.72E-02	4	0	2417
234734_s_at	ENST00000395799	TNRC6A	-0.15	2.72E-02	4	0	2420
234734_s_at	ENST00000352205	TNRC6A	-0.15	2.72E-02	4	0	2417
234734_s_at	ENST00000338120	TNRC6A	-0.15	2.72E-02	4	0	2417

209051_s_at	ENST00000393160	RALGDS	-0.15	3.74E-02	1	1	867
209051_s_at	ENST00000372062	RALGDS	-0.15	3.74E-02	1	1	867
209051_s_at	ENST00000393157	RALGDS	-0.15	3.74E-02	1	1	867
209051_s_at	ENST00000372050	RALGDS	-0.15	3.74E-02	1	1	864
209051_s_at	ENST00000372047	RALGDS	-0.15	3.74E-02	1	1	867
209051_s_at	ENST00000372051	RALGDS	-0.15	3.74E-02	1	1	1365
223571_at	ENST00000337843	C1QTNF6	-0.15	4.27E-02	1	0	2022
218596_at	ENST00000372648	TBC1D13	-0.15	3.44E-02	1	1	2545
218596_at	ENST00000223865	TBC1D13	-0.15	3.44E-02	1	1	2545
227374_at	ENST00000341597	EARS2	-0.15	1.46E-02	2	1	2264
203938_s_at	ENST00000378541	TAF1C	-0.15	6.74E-03	1	1	1086
203938_s_at	ENST00000341690	TAF1C	-0.15	6.74E-03	1	1	1086
213521_at	ENST00000347849	PTPN18	-0.15	5.02E-03	1	1	1346
213521_at	ENST00000175756	PTPN18	-0.15	5.02E-03	1	1	1346
238829_at	ENST00000261868	EIF3J	-0.15	2.76E-03	3	1	1654
204155_s_at	ENST00000375288	ENSG00000160584	-0.15	3.03E-02	1	0	2239
204155_s_at	ENST00000292055	ENSG00000160584	-0.15	3.03E-02	1	0	2239
204155_s_at	ENST00000375300	ENSG00000160584	-0.15	3.03E-02	1	0	2241
1558914_at	ENST00000346571	FAM152A	-0.15	4.12E-02	1	0	3244
203016_s_at	ENST00000342203	SSX2IP	-0.14	1.07E-02	1	1	3726
1570255_s_at	ENST00000358870	ENSG00000204716	-0.14	4.19E-02	1	0	3969
218857_s_at	ENST00000301776	ASRGL1	-0.14	2.77E-02	1	0	1122
219328_at	ENST00000372159	DDX31	-0.14	4.26E-02	1	0	577
219328_at	ENST00000372153	DDX31	-0.14	4.26E-02	1	0	577
219328_at	ENST00000372155	DDX31	-0.14	4.26E-02	1	0	2155
222433_at	ENST00000366843	ENAH	-0.14	1.96E-02	1	1	10943
222433_at	ENST00000366844	ENAH	-0.14	1.96E-02	1	1	10943
222433_at	ENST00000284563	ENAH	-0.14	1.96E-02	1	1	10943
222433_at	ENST00000391874	ENAH	-0.14	1.96E-02	1	1	10943
238054_at	ENST00000375418	ADPRHL1	-0.14	1.39E-02	1	0	875
238054_at	ENST00000356501	ADPRHL1	-0.14	1.39E-02	1	0	875
204655_at	ENST00000366113	CCL5	-0.14	1.49E-02	1	1	886
204655_at	ENST00000293272	CCL5	-0.14	1.49E-02	1	1	881
226483_at	ENST00000334667	TMEM68	-0.14	4.29E-02	1	1	1371
209334_s_at	ENST00000261817	PSMD9	-0.14	1.63E-02	2	0	1553
201802_at	ENST00000393844	SLC29A1	-0.14	1.73E-02	1	0	623
201802_at	ENST00000371755	SLC29A1	-0.14	1.73E-02	1	0	623
201802_at	ENST00000393841	SLC29A1	-0.14	1.73E-02	1	0	623
201802_at	ENST00000313248	SLC29A1	-0.14	1.73E-02	1	0	613
201802_at	ENST00000371740	SLC29A1	-0.14	1.73E-02	1	0	613
201802_at	ENST00000371731	SLC29A1	-0.14	1.73E-02	1	0	613
201802_at	ENST00000371724	SLC29A1	-0.14	1.73E-02	1	0	613
201802_at	ENST00000371713	SLC29A1	-0.14	1.73E-02	1	0	613
201802_at	ENST00000371708	SLC29A1	-0.14	1.73E-02	1	0	613
1554827_a_at	ENST00000254235	ADCY7	-0.14	3.13E-02	1	0	2619
1554827_a_at	ENST00000394697	ADCY7	-0.14	3.13E-02	1	0	2630

228857_at	ENST00000383450	ENSG00000206412	-0.14	1.99E-02	3	1	4695
228857_at	ENST00000383596	ENSG00000206492	-0.14	1.99E-02	3	1	4695
217411_s_at	ENST00000379933	RREB1	-0.13	4.22E-02	1	0	1994
217411_s_at	ENST00000349384	RREB1	-0.13	4.22E-02	1	0	1994
217411_s_at	ENST00000334984	RREB1	-0.13	4.22E-02	1	0	1994
217411_s_at	ENST00000379938	RREB1	-0.13	4.22E-02	1	0	3012
230053_at	ENST00000278505	ENDOD1	-0.13	3.44E-02	1	0	3072
228012_at	ENST00000394800	MATR3	-0.13	3.80E-02	1	1	2276
209695_at	ENST00000329397	PTP4A3	-0.13	2.69E-02	1	0	476
209695_at	ENST00000349124	PTP4A3	-0.13	2.69E-02	1	0	476
209695_at	ENST00000327627	PTP4A3	-0.13	2.69E-02	1	0	476
218553_s_at	ENST00000284006	KCTD15	-0.13	1.28E-02	2	1	2437
206034_at	ENST00000353706	SERPINB8	-0.13	3.73E-03	1	0	2093
206034_at	ENST00000397985	SERPINB8	-0.13	3.73E-03	1	0	2093
242866_x_at	ENST00000292077	POU2F2	-0.13	3.00E-02	2	0	3029
242866_x_at	ENST00000389341	POU2F2	-0.13	3.00E-02	2	0	3029
206240_s_at	ENST00000343979	ENSG00000196646	-0.13	4.89E-03	1	1	1247
227292_at	ENST00000294244	C11orf84	-0.13	4.45E-02	1	0	540
225986_x_at	ENST00000298875	CPSF2	-0.13	8.94E-04	2	1	2455
225867_at	ENST00000304735	VASN	-0.13	8.00E-06	1	1	629
1552914_a_at	ENST00000318443	CD276	-0.12	4.25E-02	2	1	1553
1552914_a_at	ENST00000318424	CD276	-0.12	4.25E-02	2	1	1553
225041_at	ENST00000361479	MPHOSPH8	-0.12	4.69E-02	1	1	623
225041_at	ENST00000360754	MPHOSPH8	-0.12	4.69E-02	1	1	691
222245_s_at	ENST00000400461	ENSG00000088340	-0.12	2.40E-02	1	0	2805
222245_s_at	ENST00000300494	ENSG00000088340	-0.12	2.40E-02	1	0	3201
220262_s_at	ENST00000357338	DLK2	-0.12	9.70E-03	1	0	185
220262_s_at	ENST00000372488	DLK2	-0.12	9.70E-03	1	0	185
220262_s_at	ENST00000372485	DLK2	-0.12	9.70E-03	1	0	187
220262_s_at	ENST00000372496	DLK2	-0.12	9.70E-03	1	0	801
217262_s_at	ENST00000262738	CELSR1	-0.12	3.22E-02	2	0	2344
202328_s_at	ENST00000382481	PKD1	-0.12	7.83E-03	2	0	4949
208072_s_at	ENST00000264057	DGKD	-0.12	2.13E-02	1	1	2637
242141_at	ENST00000398283	HDAC2	-0.12	3.46E-02	2	1	4893
64899_at	ENST00000251473	ENSG00000105520	-0.12	1.71E-02	1	0	1317
243868_at	ENST00000381992	RFX3	-0.12	3.67E-02	1	0	1920
212583_at	ENST00000156471	AQR	-0.11	4.54E-02	1	0	1243
229721_x_at	ENST00000318109	DERL3	-0.11	4.25E-02	2	0	2364
229721_x_at	ENST00000290730	DERL3	-0.11	4.25E-02	2	0	2555
218114_at	ENST00000343632	GGA1	-0.11	2.70E-02	1	1	852
218114_at	ENST00000325180	GGA1	-0.11	2.70E-02	1	1	852
218114_at	ENST00000337437	GGA1	-0.11	2.70E-02	1	1	852
210424_s_at	ENST00000360553	GOLGA8A	-0.11	8.28E-03	1	0	2343
210424_s_at	ENST00000359187	GOLGA8A	-0.11	8.28E-03	1	0	2347
210424_s_at	ENST00000341650	GOLGA8B	-0.11	8.28E-03	1	0	419
210424_s_at	ENST00000267731	GOLGA8B	-0.11	8.28E-03	1	0	1975

220813_at	ENST00000282018	CYSLTR2	-0.11	8.08E-03	1	1	1504
229912_at	ENST00000389530	SDK1	-0.11	4.29E-02	1	0	3616
229912_at	ENST00000389531	SDK1	-0.11	4.29E-02	1	0	1024
236436_at	ENST00000398802	SLC25A45	-0.11	1.78E-02	1	0	1215
236436_at	ENST00000294187	SLC25A45	-0.11	1.78E-02	1	0	1215
236436_at	ENST00000377152	SLC25A45	-0.11	1.78E-02	1	0	1215
235553_at	ENST00000394105	GAPVD1	-0.11	4.98E-02	1	0	2264
235553_at	ENST00000394104	GAPVD1	-0.11	4.98E-02	1	0	2264
235553_at	ENST00000394101	GAPVD1	-0.11	4.98E-02	1	0	4461
235553_at	ENST00000394083	GAPVD1	-0.11	4.98E-02	1	0	2264
235553_at	ENST00000265956	GAPVD1	-0.11	4.98E-02	1	0	3647
223986_x_at	ENST00000382255	DMRT2	-0.11	2.42E-02	1	0	1352
223986_x_at	ENST00000259622	DMRT2	-0.11	2.42E-02	1	0	1778
223741_s_at	ENST00000269346	TTYH2	-0.11	2.32E-02	2	0	1807
223131_s_at	ENST00000302424	TRIM8	-0.10	1.28E-03	1	0	963
223131_s_at	ENST00000369896	TRIM8	-0.10	1.28E-03	1	0	1053
205096_at	ENST00000395270	ENSG00000196313	-0.10	2.37E-02	1	0	2970
204303_s_at	ENST00000382998	KIAA0427	-0.10	3.42E-02	2	1	3648
204303_s_at	ENST00000256413	KIAA0427	-0.10	3.42E-02	2	1	3648
219190_s_at	ENST00000373210	EIF2C4	-0.10	1.24E-02	1	1	3214
219190_s_at	ENST00000397882	EIF2C4	-0.10	1.24E-02	1	1	3214
234871_at	ENST00000399043	GPR98	-0.10	4.29E-02	6	1	12263
228096_at	ENST00000322753	C1orf151	-0.09	4.58E-02	1	0	1913
232217_at	ENST00000368599	FAM26E	-0.09	3.24E-02	1	1	2556
206764_x_at	ENST00000344987	MPPE1	-0.09	4.73E-02	1	0	375
206764_x_at	ENST00000309976	MPPE1	-0.09	4.73E-02	2	0	974
206764_x_at	ENST00000317235	MPPE1	-0.09	4.73E-02	2	0	974
241910_x_at	ENST00000336125	ARL17	-0.09	4.25E-02	5	3	3298
241910_x_at	ENST00000398255	ARL17P1	-0.09	4.25E-02	5	3	3298
1555393_s_at	ENST00000397841	C21orf67	-0.09	2.23E-02	1	0	1400
204967_at	ENST00000380913	SHROOM2	-0.09	1.61E-02	1	0	2504
1556896_at	ENST00000371639	ENSG00000203999	-0.09	4.29E-02	1	0	1931
206012_at	ENST00000366820	LEFTY2	-0.09	3.62E-02	1	0	837
234676_s_at	ENST00000303775	N6AMT1	-0.09	2.69E-02	2	0	4194
234676_s_at	ENST00000351429	N6AMT1	-0.09	2.69E-02	2	0	4194
226623_at	ENST00000373880	PHYHIPL	-0.09	4.59E-02	1	1	1699
226623_at	ENST00000373878	PHYHIPL	-0.09	4.59E-02	1	1	2183
236087_at	ENST00000400045	ABLIM2	-0.09	5.64E-03	1	0	688
236087_at	ENST00000341937	ABLIM2	-0.09	5.64E-03	1	0	688
236087_at	ENST00000296372	ABLIM2	-0.09	5.64E-03	1	0	688
236087_at	ENST00000361581	ABLIM2	-0.09	5.64E-03	1	0	688
236087_at	ENST00000361737	ABLIM2	-0.09	5.64E-03	1	0	688
236087_at	ENST00000318888	ABLIM2	-0.09	5.64E-03	1	0	688
242957_at	ENST00000335613	VWCE	-0.08	3.53E-02	1	1	389
236638_at	ENST00000321420	FAM123C	-0.08	4.88E-02	1	0	3476
238437_at	ENST00000354309	ZNF805	-0.08	4.78E-02	2	0	7588

242746_at	ENST00000355818	KLHDC10	-0.08	3.57E-03	1	0	3007
228342_s_at	ENST00000258888	ALPK3	-0.08	4.01E-02	1	0	5023
213679_at	ENST00000355689	TTC30A	-0.08	3.19E-02	1	0	2406
215122_at	ENST00000321694	ZNF688	-0.07	4.26E-02	1	0	1290
229929_at	ENST00000393014	SPSB4	-0.07	4.30E-02	1	1	1341
229929_at	ENST00000310546	SPSB4	-0.07	4.30E-02	1	1	1341
1552410_at	ENST00000272367	CLEC4F	-0.07	4.19E-03	1	0	621
233437_at	ENST00000264318	GABRA4	-0.07	5.25E-03	1	0	9323
203568_s_at	ENST00000349458	TRIM38	-0.06	4.26E-02	1	0	1433
203568_s_at	ENST00000357085	TRIM38	-0.06	4.26E-02	1	0	1383
206780_at	ENST00000259271	GAD2	-0.06	3.44E-02	1	0	559
206780_at	ENST00000376261	GAD2	-0.06	3.44E-02	2	0	3597
224704_at	ENST00000395801	TNRC6A	-0.06	2.12E-02	4	0	2417
224704_at	ENST00000395799	TNRC6A	-0.06	2.12E-02	4	0	2420
224704_at	ENST00000352205	TNRC6A	-0.06	2.12E-02	4	0	2417
224704_at	ENST00000338120	TNRC6A	-0.06	2.12E-02	4	0	2417
221667_s_at	ENST00000281938	HSPB8	-0.05	4.34E-02	1	0	887
201869_s_at	ENST00000217964	TBL1X	-0.05	3.20E-02	2	0	3491
238613_at	ENST00000338983	ENSG00000091436	-0.05	1.10E-02	2	0	5613
208253_at	ENST00000340550	SIGLEC8	-0.05	2.41E-02	1	0	1381
208253_at	ENST00000321424	SIGLEC8	-0.05	2.41E-02	1	0	1381
208169_s_at	ENST00000354608	PTGER3	-0.03	1.21E-02	1	1	699
212707_s_at	ENST00000262940	RASA4	-0.03	2.88E-02	1	0	3099
213809_x_at	ENST00000344749	TCF3	-0.02	2.74E-02	1	0	2413
